# Supplementary material for: Tetherin antagonism by SARS‐CoV‐2 ORF3a and spike protein enhances virus release
Source: EMBO Rep. 2023 Oct 11;24(12):e57224. doi: 10.15252/embr.202357224 (PMC10702813; doi:10.15252/embr.202357224)
Supplement: Supplementary file 1 — Appendix S1 [file EMBR-24-e57224-s001.pdf]

Appendix

Table of Contents

Appendix Figure S1 2

Appendix Figure S2 3

Appendix Figure S1

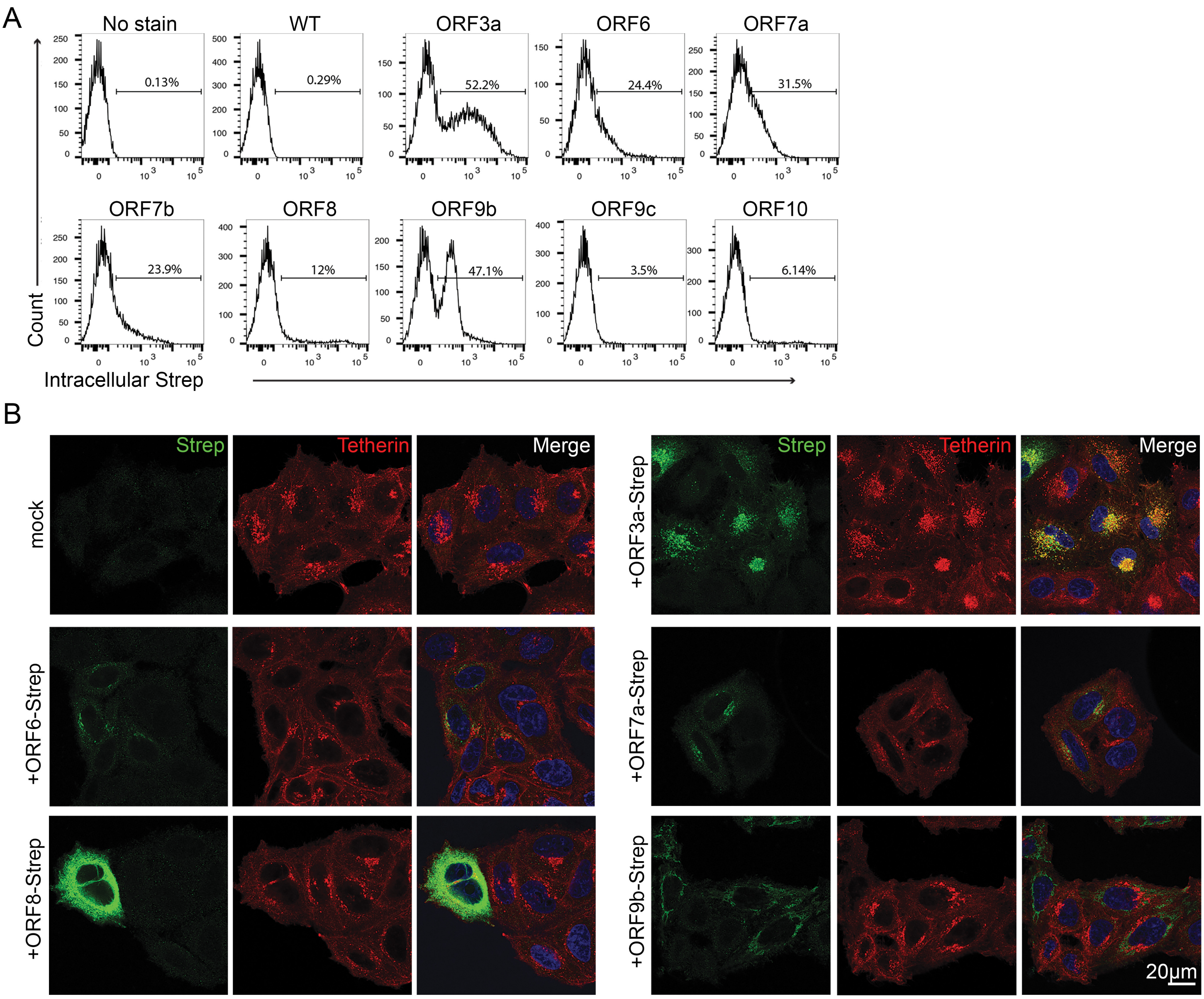

SARS-CoV-2 ORF miniscreen highlights ORF3a in altering tetherin disribution

(A) SARS-CoV-2 ORFs were transiently expressed in HeLa cells. Cells were fixed, permeabilized and stained with anti-Strep antibodies and analysed by flow cytometry to confirm cells expressing strep-tagged SARS-CoV-2 ORFs.

(B) Representative confocal immunofluorescence microscopy images of fixed HeLa cells transiently transfected with SARS-CoV-2 ORFs. Anti-Strep (green), anti-tetherin (red), DAPI (blue). Scale bar, 20 µm.

Appendix Figure S2

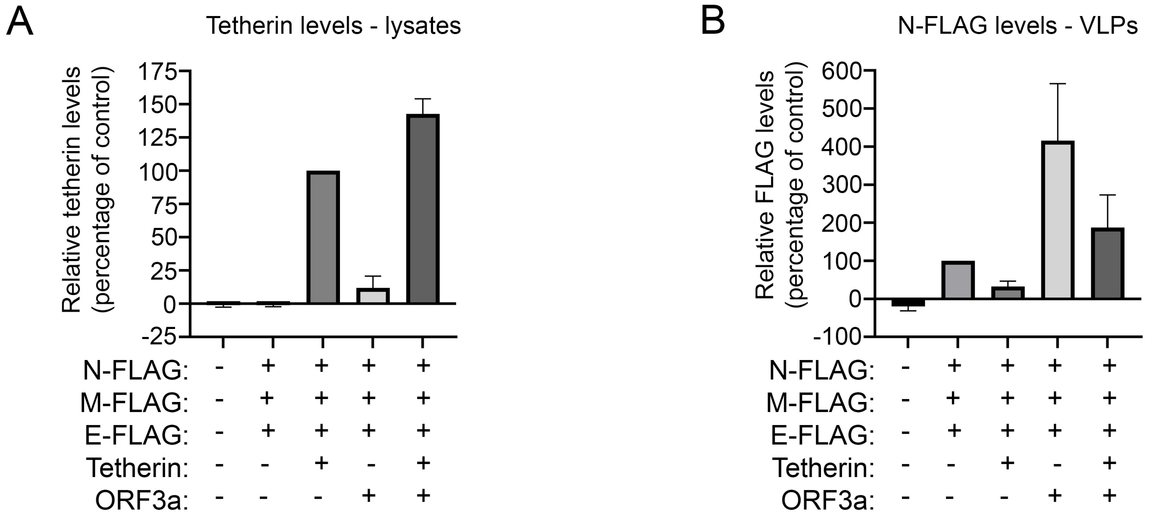

SARS-CoV-2 ORF3a enhances VLP release.

(A) Western blot densitometry analysis of HEK293T cells transiently transfected with FLAG-tagged SARS-CoV-2 structural proteins (M/E/N), tetherin and ORF3a (see Figure 6A). Tetherin abundance was analysed in cell lysates. Values are normalised to +M/E/N +Tetherin cells (lane 3). Mock -1.1% (SD: 2.6%), +M/E/N -1.1% (SD: 2.1%), +M/E/N +Tetherin 100%, +M/E/N +ORF3a 11.9% (SD 15.4%), +M/E/N +Tetherin +ORF3a 142.4% (SD: 20.3%). The mean and standard deviation are shown. Data from three biological replicates.

(B) Western blot densitometry analysis of VLPs from HEK293T cells transiently transfected with FLAG-tagged SARS-CoV-2 structural proteins (M/E/N), tetherin and ORF3a (see Figure 6A). N-FLAG abundance was analysed from VLPs. Values are normalised to +VLPs from M/E/N cells (lane 2). Mock -19.8% (SD: 20.2%), +M/E/N 100%, +M/E/N +Tetherin 32.7% (SD:23.7%), +M/E/N +ORF3a 425.9% (SD: 260.6%), +M/E/N +Tetherin +ORF3a 187.6% (SD 147.4%). The mean and standard deviation are shown. Data from three biological replicates.
