# Supplementary material for: Tetherin antagonism by SARS‐CoV‐2 ORF3a and spike protein enhances virus release
Source: EMBO Rep. 2023 Oct 11;24(12):e57224. doi: 10.15252/embr.202357224 (PMC10702813; doi:10.15252/embr.202357224)
Supplement: Supplementary file 2 — Expanded View Figures PDF [file EMBR-24-e57224-s009.pdf]

Expanded View Figures

Figure EV1. SARS-CoV-2 infection downregulates tetherin in primary human airway epithelial, and in immortalised HeLa + ACE2, A549 + ACE2 and T84 cells.

A Differentiated nasal primary human airway epithelial (HAE) cells were embedded to OCT. Cryostat sections were stained for spike (green), tetherin (red), phalloidin (grey) and DAPI (blue).

B Differentiated nasal primary human airway epithelial (HAE) cells were infected with SARS-CoV-2 (MOI 0.01). Cells were fixed at 48 hpi and embedded to OCT. Cryostat sections were collected and prepared for confocal microscopy. Sections were immunolabelled with antibodies against SARS-CoV-2 spike (green)—to reveal SARS-CoV-2 infected cells, tetherin (red), and with phalloidin-647 (grey) and DAPI (blue).

C The mean fluorescence intensity of tetherin was quantified in Spike negative (uninfected) and Spike positive (infected) HAE cells infected with SARS-CoV-2 (as in Figs 1A and EV1B) and values are normalised to uninfected HAE cells. Spike negative: 100% (SD: 38.4%), 26 cells; Spike positive: 66.7% (SD: 48.3%), 15 cells. Data from two biological replicates. Individual data points, mean and standard deviation are shown.

D SARS-CoV-2 infected HeLa + ACE2 cells (MOI 0.5) were fixed at 24 hpi and stained for spike (green) and tetherin (red). Infected cells display reduced tetherin levels, and broad loss of tetherin from the plasma membrane. Where cell surface tetherin remains, it is often clustered with spike staining (enlarged, arrows). Uninfected cells shown with asterisk.

E The mean fluorescence intensity of tetherin was quantified in Spike negative (uninfected) and Spike positive (infected) HeLa + ACE2 cells (as in Figs 1D and EV1D) and values are normalised to uninfected HeLa cells. Spike negative: 100% (SD: 22.2%), 27 cells; Spike positive: 52.6% (SD: 21.0%), 15 cells. Data from three technical and two biological replicates. Individual data points, mean and standard deviation are shown.

F HeLa + ACE2 cells were infected with SARS-CoV-2 (MOI 0.5) and fixed at 24 hpi. Cells were stained for spike (green) and beta2microglobulin (red). No differences in beta2microglobulin were observed between infected and uninfected cells.

G Tetherin expression is induced with IFNα in A549 cells. Mock and IFNα treated (1,000 U/ml, 24 h) A549 cells were fixed and stained for tetherin (red) and analysed by confocal immunofluorescence microscopy.

H The mean fluorescence intensity of tetherin was quantified in uninfected (Spike negative) or SARS-CoV-2 infected A549 + ACE2 cells (Spike positive; see Fig 2C). Values are normalised to the mean fluorescence intensity of uninfected A549 + ACE2 cells. Spike negative: 100% (SD: 39.5%), 22 cells; Spike positive: 35.2% (SD: 12.2%), 16 cells. Data from three technical and two biological replicates. Individual data points, mean and standard deviation are shown.

I A549 + ACE2 cells were treated with IFNα (1,000 U/ml) and infected with SARS-CoV-2 (MOI 0.5), fixed at 24 hpi and processed for TEM. DMVs (some, but not all) are highlighted with asterisks.

J The mean fluorescence intensity of tetherin was quantified in uninfected (Spike negative) or SARS-CoV-2 infected T84 cells (Spike positive; see Fig 2G). Values are normalised to the mean fluorescence intensity of uninfected A549 + ACE2 cells. The mean and standard deviation are shown. Spike negative: 100% (SD: 49.9%), 22 cells; Spike positive: 39.7% (SD: 14.9%), 14 cells. Data from three technical and two biological replicates. Individual data points, mean and standard deviation are shown.

K Additional electron micrographs of SARS-CoV-2 virions in infected T84 cells. T84 cells were infected with SARS-CoV-2 (MOI 0.5) and fixed at 24 hpi. Tethered virions were frequently present at the plasma membrane (highlighted by arrowheads), and in intracellular compartments.

© 2023 The Authors

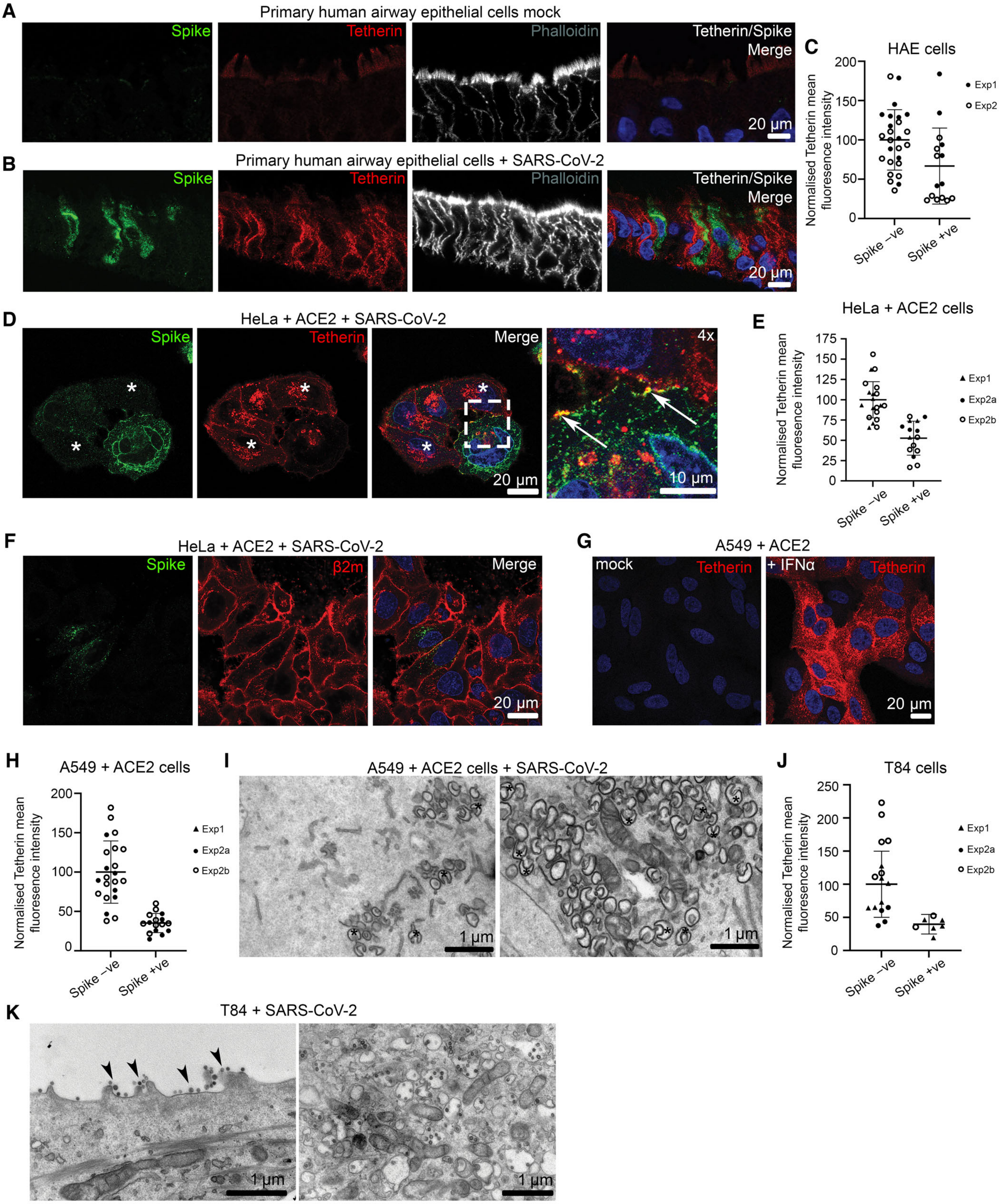
A
Primary human airway epithelial cells mock
Spike
Tetherin
Phalloidin
Tetherin/Spike Merge
20 µm
B
Primary human airway epithelial cells + SARS-CoV-2
Spike
Tetherin
Phalloidin
Tetherin/Spike Merge
20 µm
C
HAE cells
Normalised Tetherin mean fluoresence intensity
200
150
100
50
0
Exp1
Exp2
Spike –ve
Spike +ve
D
HeLa + ACE2 + SARS-CoV-2
Spike
Tetherin
Merge
4x
20 µm
10 µm
E
HeLa + ACE2 cells
Normalised Tetherin mean fluoresence intensity
175
150
125
100
75
50
25
0
Exp1
Exp2a
Exp2b
Spike –ve
Spike +ve
F
HeLa + ACE2 + SARS-CoV-2
Spike
β2m
Merge
20 µm
G
A549 + ACE2
mock
Tetherin
+ IFNα
Tetherin
20 µm
H
A549 + ACE2 cells
Normalised Tetherin mean fluoresence intensity
200
150
100
50
0
Exp1
Exp2a
Exp2b
Spike –ve
Spike +ve
I
A549 + ACE2 cells + SARS-CoV-2
1 µm
1 µm
J
T84 cells
Normalised Tetherin mean fluoresence intensity
250
200
150
100
50
0
Exp1
Exp2a
Exp2b
Spike –ve
Spike +ve
K
T84 + SARS-CoV-2
1 µm
1 µm

Figure EV1.

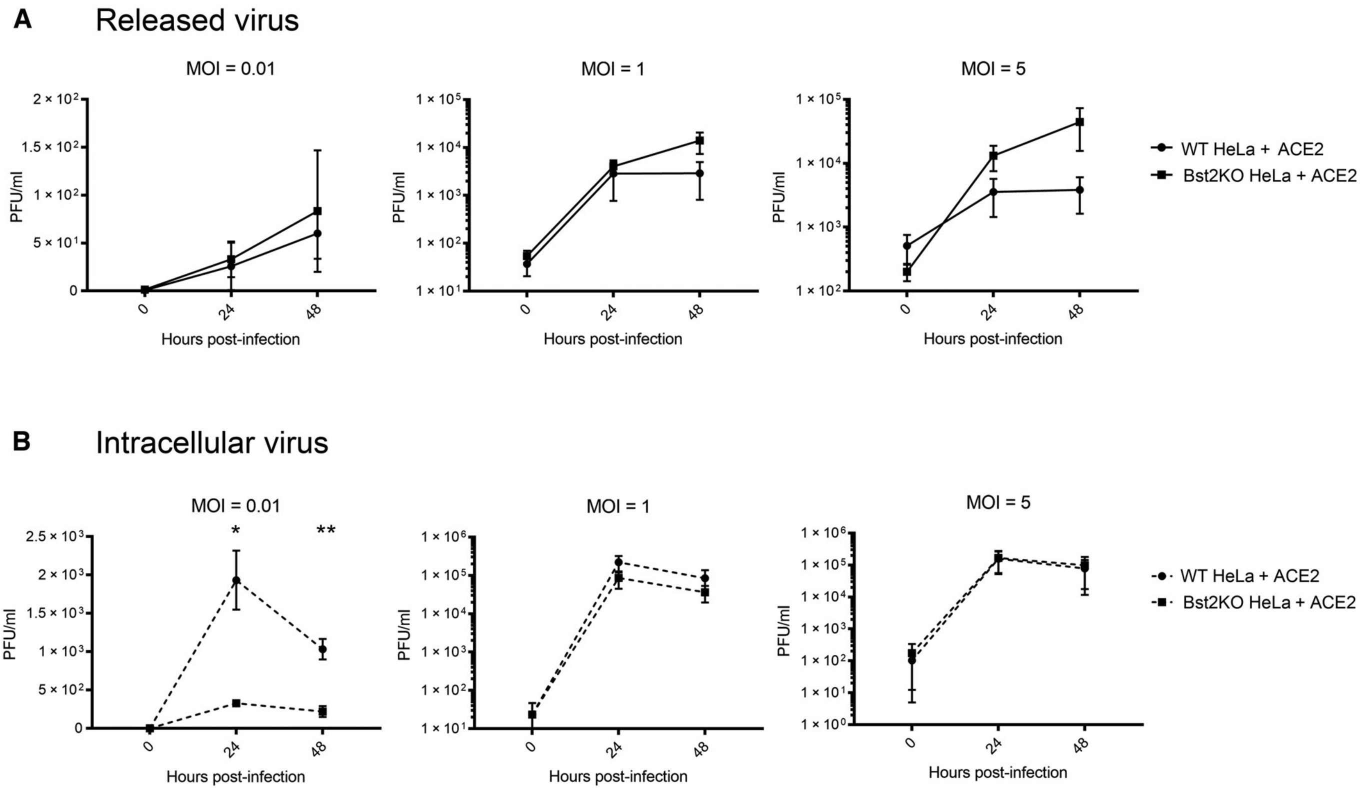

Figure EV2. Viral growth curves confirm a role for tetherin in SARS-CoV-2 release.

A Viral growth curves were performed by infecting WT HeLa + ACE2 and Bst2KO HeLa + ACE2 cells with SARS-CoV-2 at the indicated MOI (0.01, 1 or 5). Samples were taken at 0, 24 and 48 h post infection. Released viral titres were measured by plaque assays. Data represents the mean ± SEM of three independent biological replicates.

B Viral growth curves were performed by infecting WT HeLa + ACE2 and Bst2KO HeLa + ACE2 cells with SARS-CoV-2 at the indicated MOI (0.01, 1 or 5). Samples were taken at 0, 24 and 48 h post infection. Intracellular viral titres were measured by plaque assays. Data represents the mean ± SEM of three independent biological replicates. Statistical significance (*ns* [not significant] $P > 0.05$, *$P < 0.05$, **$P < 0.01$) was determined using multiple *t*-tests and the Holm–Sidak method ($\alpha = 0.05$). Each time point was analysed individually, without assuming a consistent SD. Analysis was conducted using Prism 9 (GraphPad).

Figure EV3. SARS-CoV-2 ORF7a localises to the trans-Golgi network and additional cytosolic puncta.

A Representative confocal immunofluorescence images of HeLa cells transiently transfected with SARS-CoV-1 ORF7a-FLAG or SARS-CoV-2 ORF7a-FLAG. SARS-CoV-1 ORF7a-FLAG predominately colocalizes with TGN46 (red), whilst SARS-CoV-2 ORF7a-FLAG shows additional staining outside that colocalizing with TGN46 (arrowheads).

B Manders' coefficients were calculated to measure the ORF7a-FLAG overlap with TGN46. At least 54 cells per condition from three biologically independent experiments were analysed. All data points, mean and standard deviation are plotted. Two-tailed, unpaired *t*-tests were performed (****$P < 0.0001$).

C SARS-CoV-1 ORF7a-FLAG and SARS-CoV-2 ORF7a-FLAG stable cell lines were labelled with antibodies against FLAG (green), or the trans-Golgi marker TGN46 (red), and DAPI (blue).

D As in (C) but with the cis-Golgi marker ZFPL1 (red).

E SARS-CoV-2 infected HeLa + ACE2 cells display fragmentation of Golgi markers. HeLa + ACE2 cells were infected with SARS-CoV-2 (MOI 0.5) and fixed at 24 hpi. Infected cells were identified by Spike staining (green) and cells were costained with Golgi markers TGN46 (top) and ZFPL1 (below). Areas of TGN46 and ZFPL1 are enlarged (right) to highlight Golgi fragmentation in SARS-CoV-2 infected cells.

▶

 © 2023 The Authors

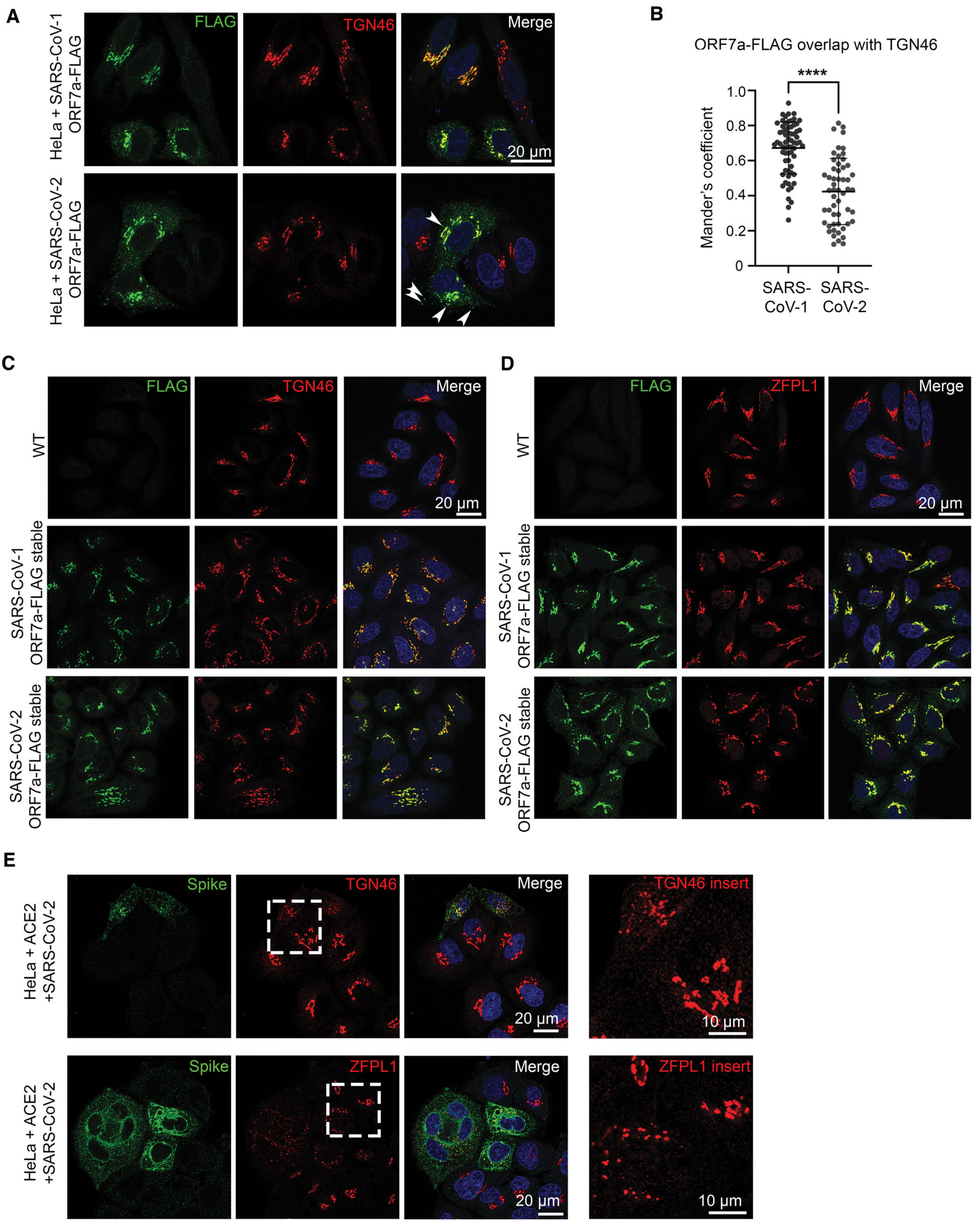
A
FLAG
TGN46
Merge
HeLa + SARS-CoV-1 ORF7a-FLAG
20 µm
HeLa + SARS-CoV-2 ORF7a-FLAG
B
ORF7a-FLAG overlap with TGN46
****
Mander's coefficient
1.0
0.8
0.6
0.4
0.2
0
SARS-CoV-1
SARS-CoV-2
C
FLAG
TGN46
Merge
WT
20 µm
SARS-CoV-1 ORF7a-FLAG stable
SARS-CoV-2 ORF7a-FLAG stable
D
FLAG
ZFPL1
Merge
WT
20 µm
SARS-CoV-1 ORF7a-FLAG stable
SARS-CoV-2 ORF7a-FLAG stable
E
Spike
TGN46
Merge
TGN46 insert
HeLa + ACE2 +SARS-CoV-2
20 µm
10 µm
Spike
ZFPL1
Merge
ZFPL1 insert
HeLa + ACE2 +SARS-CoV-2
20 µm
10 µm

Figure EV3.

Figure EV4. Spike expression causes tetherin downregulation.

A HeLa cells were transiently transfected with plasmids encoding ss-HA-Spike. 48 h post transfection, cells were fixed and stained with antibodies against anti-HA (green) and anti-tetherin (red), and DAPI (blue).

B HeLa cells were transiently transfected with ss-HA-Spike plasmids (See Fig EV4A). The mean fluorescence intensity of tetherin was quantified in HA negative or HA positive cells. Values are normalised to the mean fluorescence intensity of HA negative cells. HA negative: 100% (SD: 13.2%), 33 cells; HA positive: 70.4% (SD: 12.0%), 26 cells. Data from three technical and two biological replicates. Individual data points, mean and standard deviation are shown.

C HEK-293T cells were transfected with the indicated constructs and their surface levels determined by flow cytometry using an antibody directed against the S2 subunit of the Spike protein. The light grey trace shows the non-transfected, unstained control and the dark grey trace the sample expressing the Spike construct. The mean fluorescent intensity was calculated for each sample and plotted and error bars show the standard deviation. Data from three technical repeats.

D HEK-293T cells were transfected with the indicated constructs and incubated with tissue culture supernatant containing soluble ACE2 (ACE2-Fc) and the amount of ACE2 binding determined by flow cytometry. The light grey trace shows the non-transfected control sample and the dark grey trace the sample expressing the Spike construct. The mean fluorescent intensity was calculated for each sample and plotted and error bars show the standard deviation. Data from three technical repeats.

E HeLa + TetOne ss-HA-Spike (stable) cells were treated −/+ Doxycycline for 48 h (see Fig 4D). The mean fluorescence intensity of tetherin was quantified in cells treated with or without Doxycycline. Values are normalised to the mean fluorescence intensity of −Doxycycline cells. The mean and standard deviation are shown. −Dox: 100% (SD: 17.4%), 34 cells; +Dox: 66.6.% (SD: 16.8%), 38 cells. Data from three technical and two biological replicates. Individual data points, mean and standard deviation are shown.

F TetOne ss-HA-Spike cells were incubated with Doxycycline for 48 h. Cells were fixed and stained for HA (green), tetherin (red) and with DAPI (blue) to show the presence of multinucleated cells.

G Western blot densitometry analysis of tetherin in HeLa + TetOne ss-HA-Spike stable cells, treated −/+ Doxcycline (see Fig 4F). Tetherin abundance was analysed in cell lysates. Values are normalised to −Dox cells. The mean and standard deviation are shown. −Dox: 100%; +Dox 74.7% (SD: 8.2%). Data from three biological replicates. Two-tailed, unpaired *t*-tests were performed (*$P < 0.05$).

H Flow cytometry was performed on mock or Doxycycline treated (48 h) TetOne ss-HA-Spike cells to analyse surface CD71 levels. $n = 2$ experiments, representative data shown.

I Western blot densitometry analysis of HEK293T cells transiently transfected with FLAG-tagged SARS-CoV-2 structural proteins (M/E/N), tetherin and Spike (see Fig 4G). Tetherin abundance was analysed in cell lysates. Values are normalised to +M/E/N + Tetherin cells (lane 3). Mock −0.1% (SD: 0.11%), +M/E/N 3.5% (SD: 0.32%), +M/E/N + Tetherin 100%, +M/E/N + Tetherin + Spike 81.1% (SD 15.0%). The mean and standard deviation are shown. Data from three biological replicates.

J Western blot densitometry analysis of VLPs from HEK293T cells transiently transfected with FLAG-tagged SARS-CoV-2 structural proteins (M/E/N), tetherin and Spike (see Fig 4G). N-FLAG abundance was analysed from VLPs. Values are normalised to +VLPs from M/E/N cells (lane 2). Mock 7.3% (SD: 37.4%), +M/E/N 100%, +M/E/N + Tetherin 26.0% (SD:23.5%), +M/E/N + Tetherin + Spike 3.0% (SD 47.9%). The mean and standard deviation are shown. Data from three biological replicates.

© 2023 The Authors

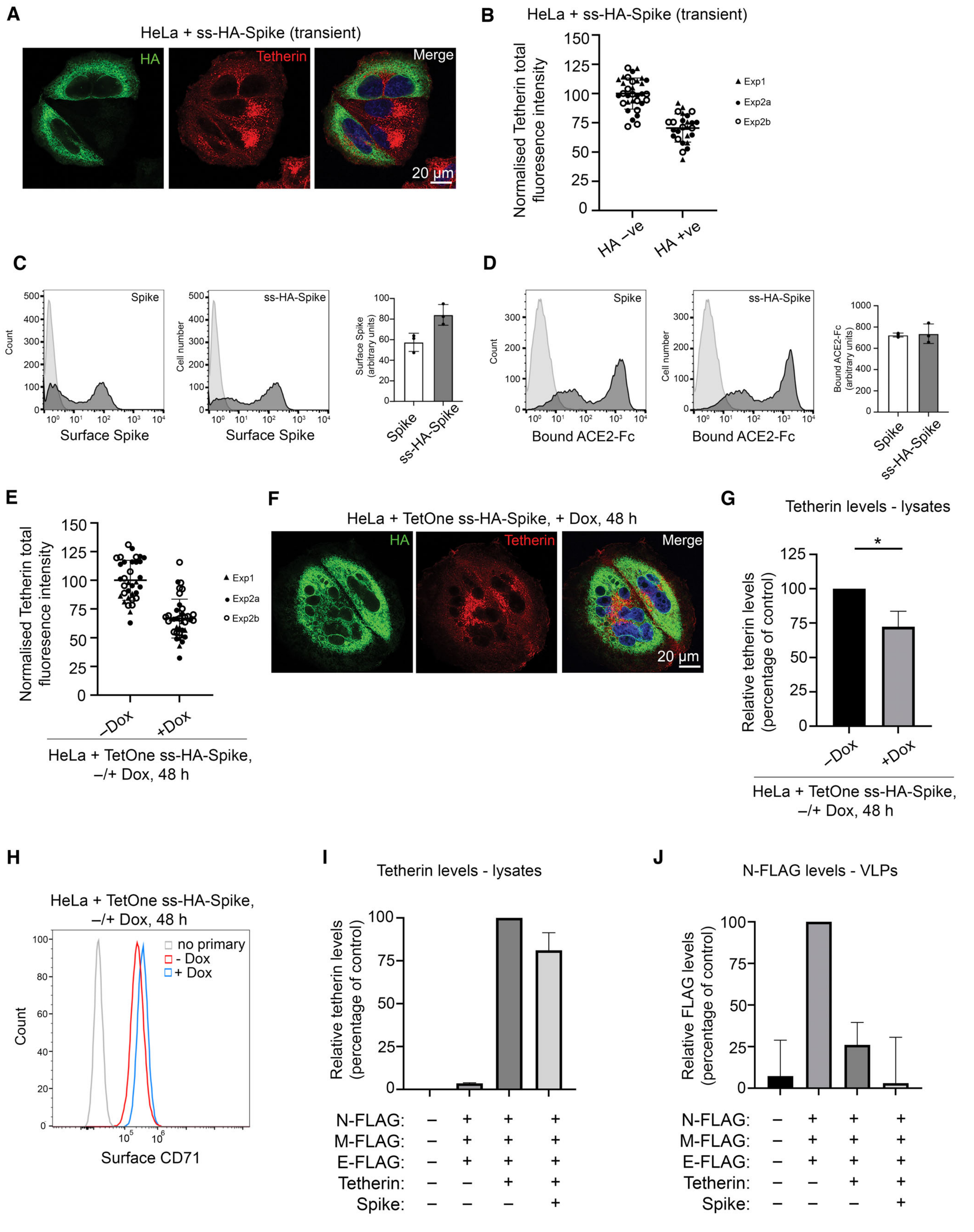
A
HeLa + ss-HA-Spike (transient)
HA
Tetherin
Merge
20 µm
B
HeLa + ss-HA-Spike (transient)
Normalised Tetherin total fluoresence intensity
Exp1
Exp2a
Exp2b
HA –ve
HA +ve
C
Spike
ss-HA-Spike
Count
Cell number
Surface Spike
Surface Spike (arbitrary units)
D
Spike
ss-HA-Spike
Count
Cell number
Bound ACE2-Fc
Bound ACE2-Fc (arbitrary units)
E
Normalised Tetherin total fluoresence intensity
Exp1
Exp2a
Exp2b
–Dox
+Dox
HeLa + TetOne ss-HA-Spike, –/+ Dox, 48 h
F
HeLa + TetOne ss-HA-Spike, + Dox, 48 h
HA
Tetherin
Merge
20 µm
G
Tetherin levels - lysates
Relative tetherin levels (percentage of control)
–Dox
+Dox
HeLa + TetOne ss-HA-Spike, –/+ Dox, 48 h
H
HeLa + TetOne ss-HA-Spike, –/+ Dox, 48 h
no primary
- Dox
+ Dox
Count
Surface CD71
I
Tetherin levels - lysates
Relative tetherin levels (percentage of control)
N-FLAG:
M-FLAG:
E-FLAG:
Tetherin:
Spike:
J
N-FLAG levels - VLPs
Relative FLAG levels (percentage of control)
N-FLAG:
M-FLAG:
E-FLAG:
Tetherin:
Spike:

Figure EV4.

Figure EV5. SARS-CoV-2 ORF3a alters endosome morphology and impairs retrograde retrieval.

A Intracellular flow cytometry was performed on mock HeLa and transiently transfected ORF3a-Strep HeLa cells to identify Strep positive (transfected) cells. The intracellular levels of tetherin were compared between the Strep negative (untransfected) and Strep positive (transfected) cells.

B Confocal immunofluorescence microscopy was performed on mock or ORF3a-Strep transfected cells to analyse the distribution of markers CIMPR, VPS35 and CD63.

C Confocal immunofluorescence microscopy was performed on mock or ORF3a-Strep transiently transfected cells to analyse the distribution of the early and late endolysosomal markers, EEA1 and Cathepsin D. Colocalisation between EEA1 and Cathepsin D could only be observed in ORF3a-Strep transfected cells. Enlarged area shows Cathepsin D within EEA1-positive compartments.

D Transmission electron microscopy was performed on mock or ORF3a-Strep transiently transfected HeLa cells. Endosomes and lysosomes appeared enlarged and containing non-resolved content in ORF3a-Strep transfected cells.

E Antibody uptake experiments were performed to follow the fate of endocytosed tetherin. ORF3a-Strep transient transfections were performed 48 h prior to uptake experiments. Anti-tetherin antibodies were bound to live cells on ice for 30 min before a 2 h chase at 37°C. Cells were fixed and immunolabelled using anti-LAMP1 (green), secondary anti-rabbit555 (red) antibodies, and DAPI (blue). Representative images are shown.

F Antibody uptake experiments were performed using anti-CIMPR antibodies. ORF3a-Strep transient transfections were performed 48 h prior to uptake experiments. Anti-CIMPR antibodies (2G11 clone) were bound to live cells on ice for 30 min before a 2 h chase at 37°C. Cells were fixed and immunolabelled using anti-TGN46 (red), secondary anti-mouse (green) antibodies, and DAPI (blue). Representative images are shown.

G Colocalisation analysis was performed to quantify the Mander's overlap coefficient of endocytosed anti-CIMPR overlapping TGN46. At least 20 cells per condition from three biological replicates were analysed. Individual data points are plotted with mean and standard deviation. Two-tailed, unpaired *t*-tests were performed (****$P < 0.0001$).

© 2023 The Authors

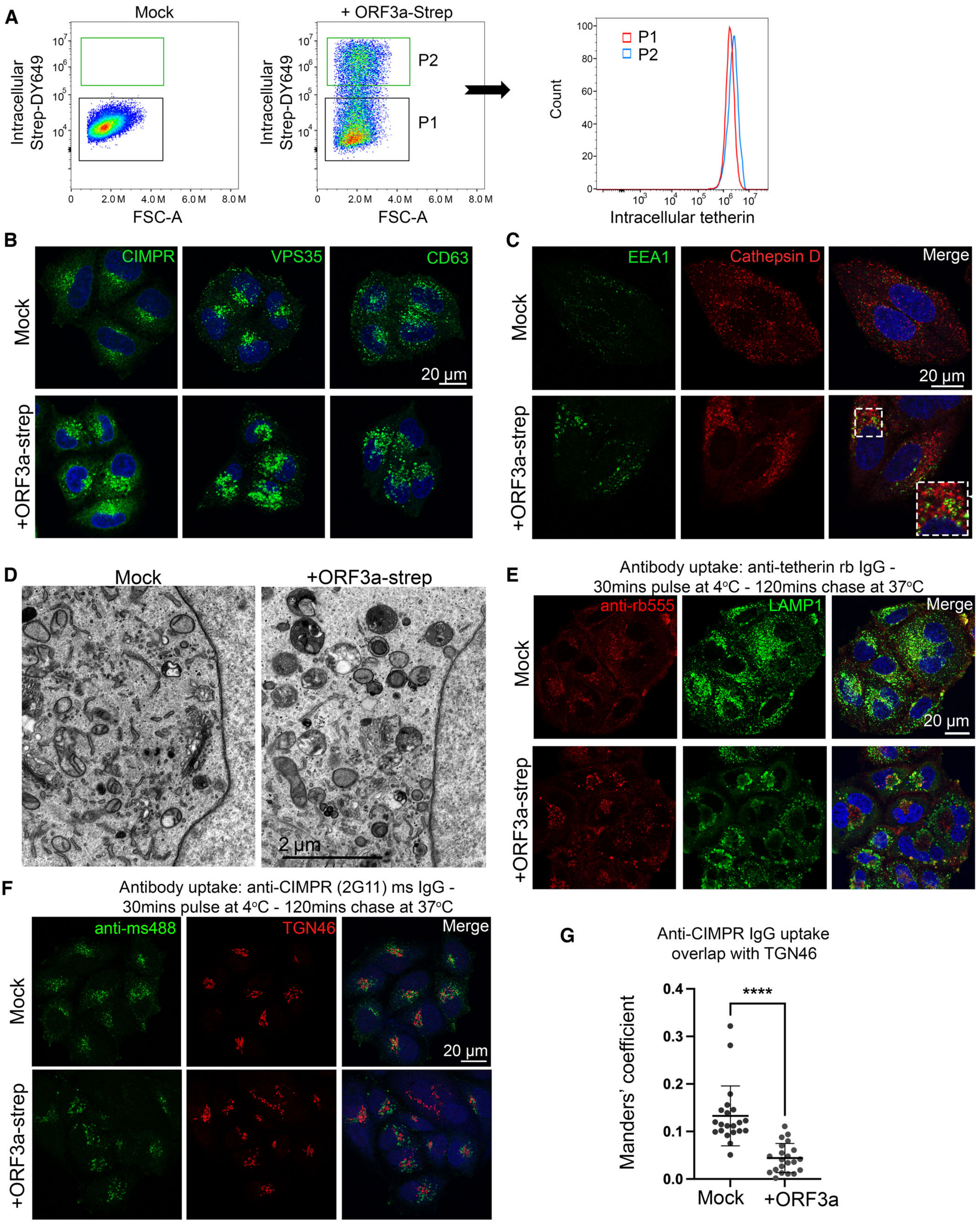
A
Mock
+ ORF3a-Strep
Intracellular Strep-DY649
FSC-A
P1
P2
Count
Intracellular tetherin
B
CIMPR
VPS35
CD63
Mock
+ORF3a-strep
20 μm
C
EEA1
Cathepsin D
Merge
D
Mock
+ORF3a-strep
2 μm
E
Antibody uptake: anti-tetherin rb IgG - 30mins pulse at 4°C - 120mins chase at 37°C
anti-rb555
LAMP1
F
Antibody uptake: anti-CIMPR (2G11) ms IgG - 30mins pulse at 4°C - 120mins chase at 37°C
anti-ms488
TGN46
G
Anti-CIMPR IgG uptake overlap with TGN46
Manders' coefficient
****
Mock
+ORF3a

Figure EV5.
