# Supplementary figures and images for: Tetherin antagonism by SARS‐CoV‐2 ORF3a and spike protein enhances virus release
Source: EMBO Rep. 2023 Oct 11;24(12):e57224. doi: 10.15252/embr.202357224 (PMC10702813; doi:10.15252/embr.202357224)

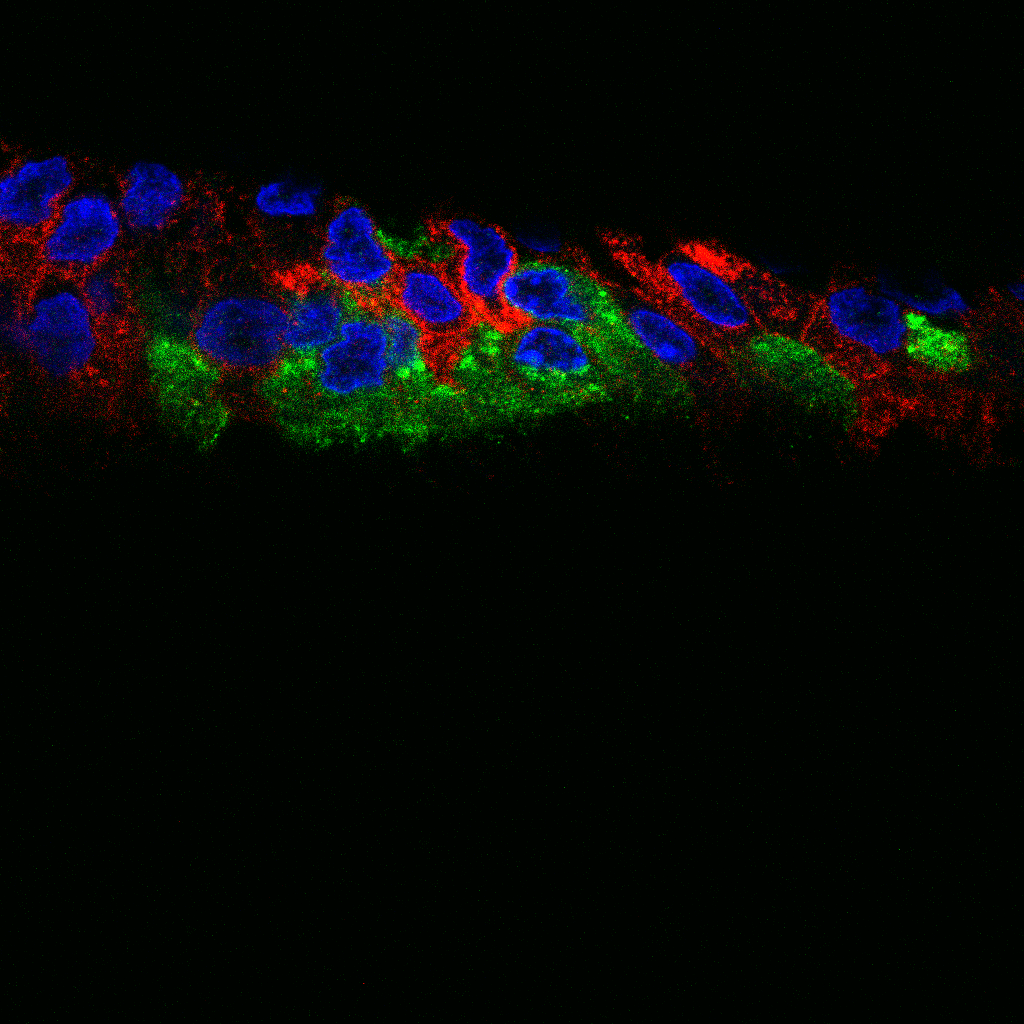

Supplement: Supplementary file 5 — Source Data for Figure 1 [file EMBR-24-e57224-s010.zip › SD Figure 1/1A/RGB merge.tif]

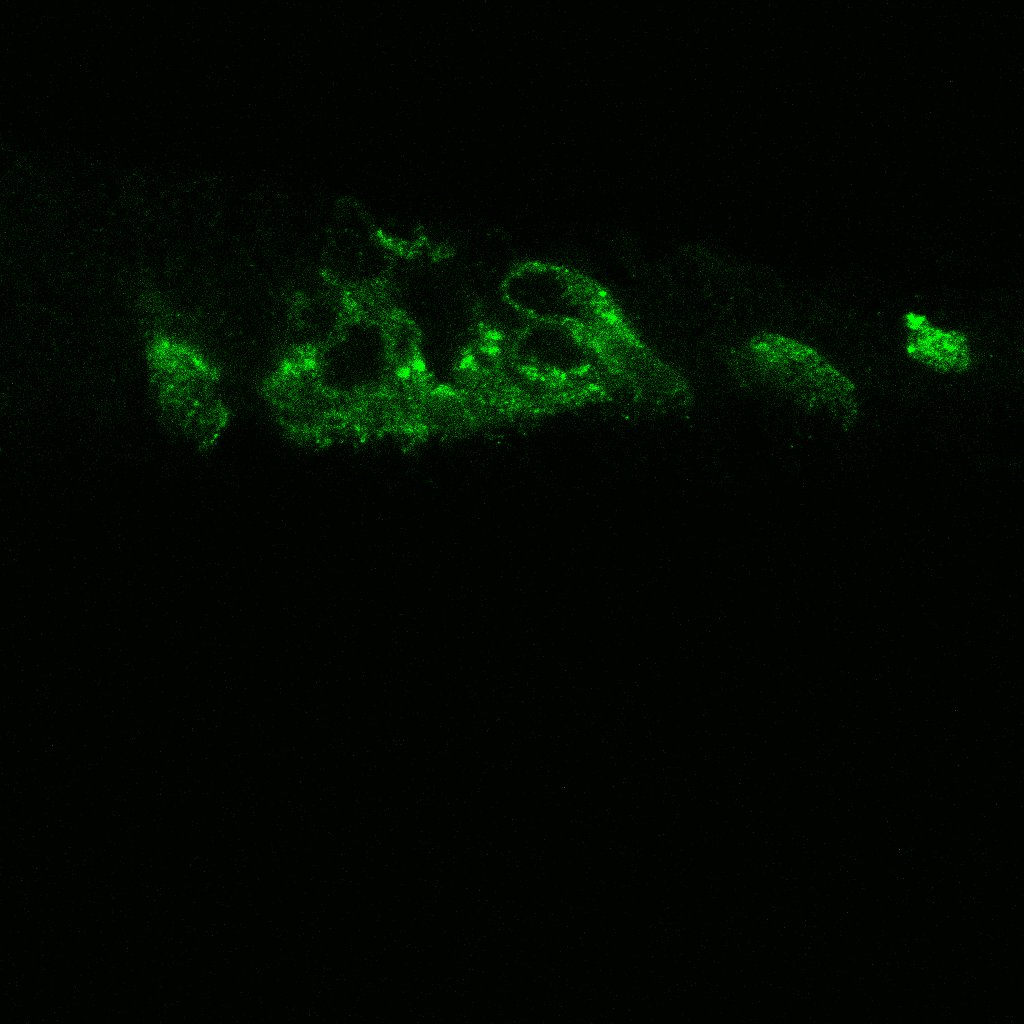

Supplement: Supplementary file 5 — Source Data for Figure 1 [file EMBR-24-e57224-s010.zip › SD Figure 1/1A/1A Green.tif]

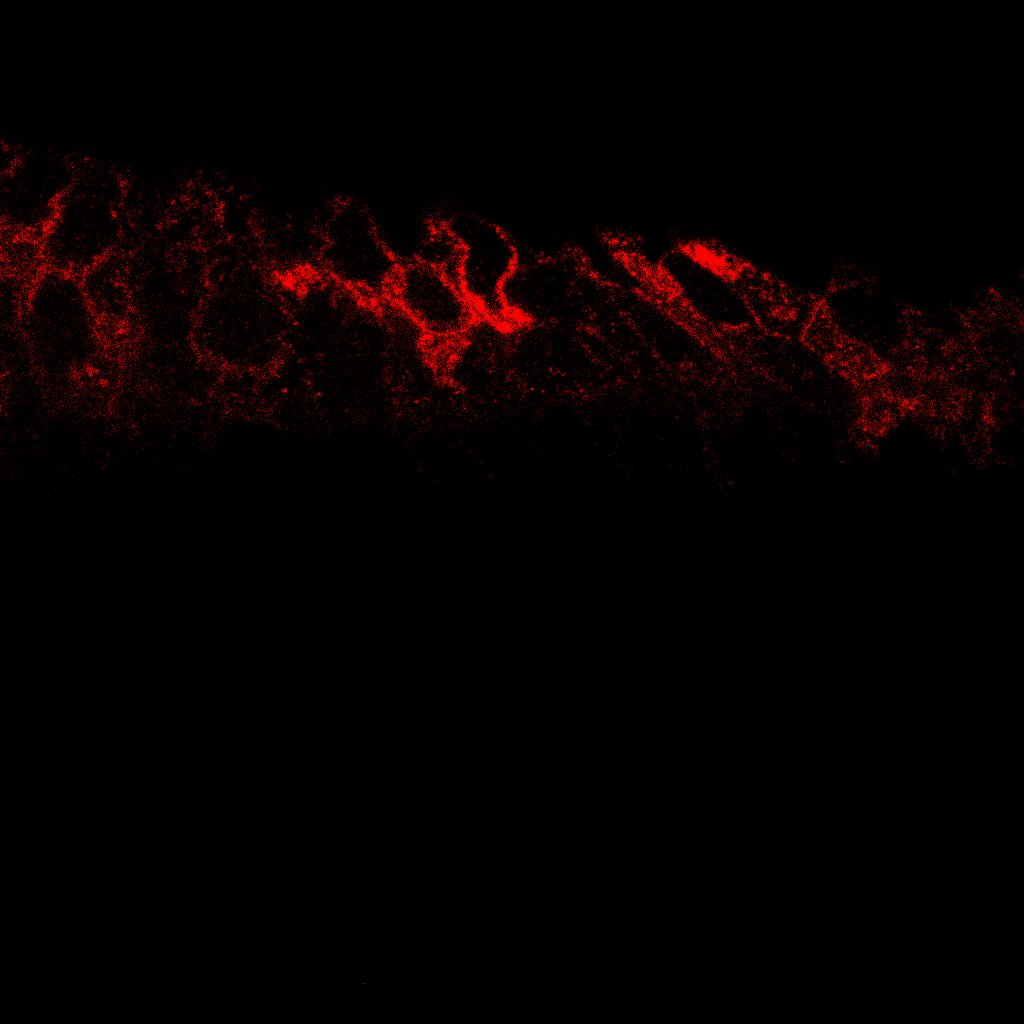

Supplement: Supplementary file 5 — Source Data for Figure 1 [file EMBR-24-e57224-s010.zip › SD Figure 1/1A/1A Red.tif]

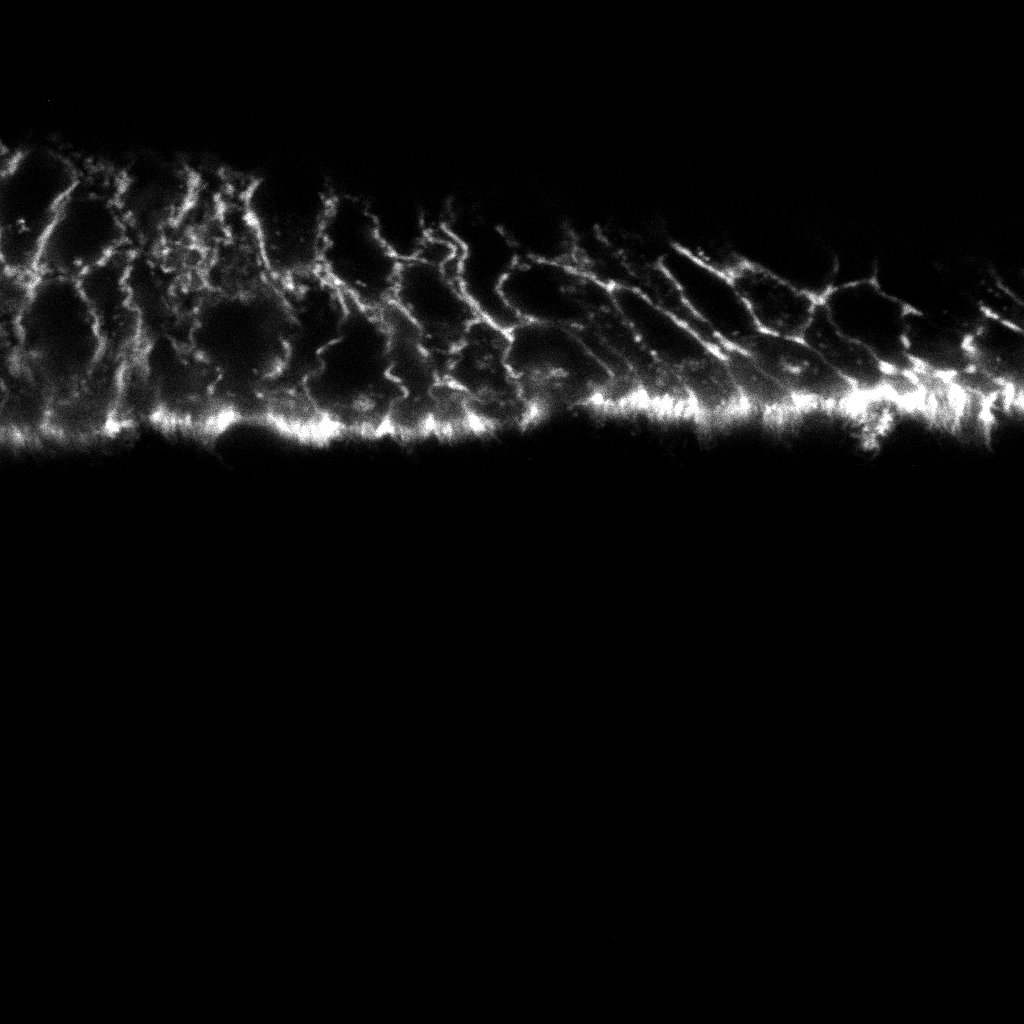

Supplement: Supplementary file 5 — Source Data for Figure 1 [file EMBR-24-e57224-s010.zip › SD Figure 1/1A/1A white.tif]

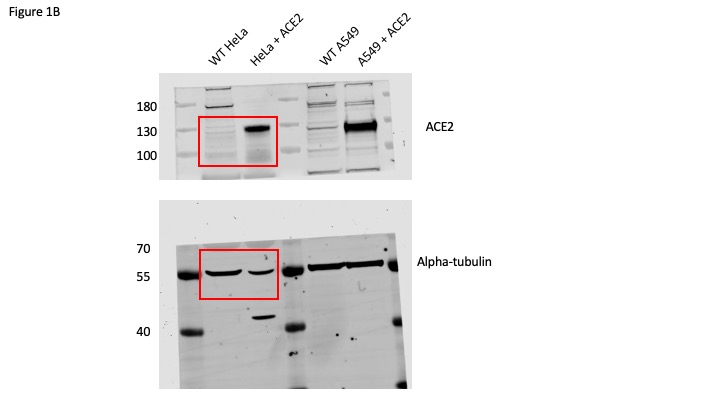

Supplement: Supplementary file 5 — Source Data for Figure 1 [file EMBR-24-e57224-s010.zip › SD Figure 1/1B/Figure 1B.jpeg]

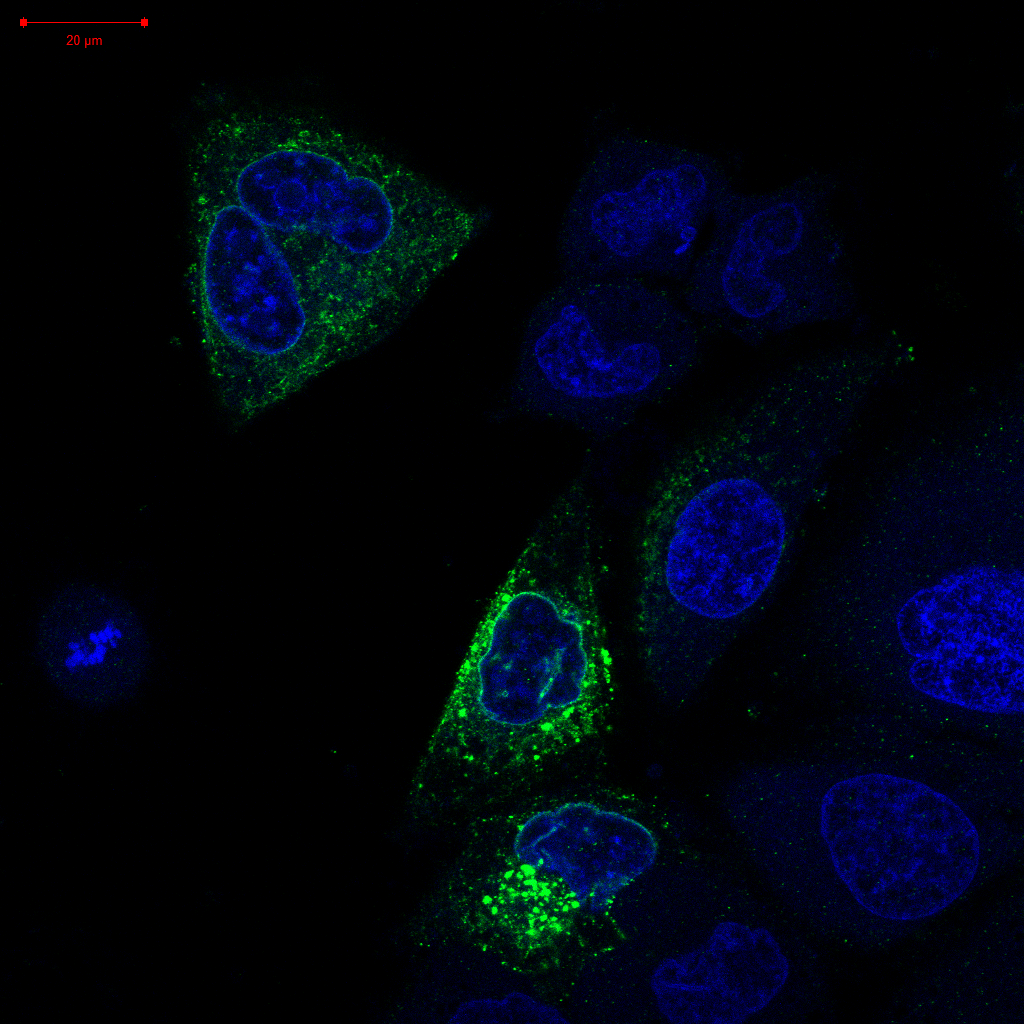

Supplement: Supplementary file 5 — Source Data for Figure 1 [file EMBR-24-e57224-s010.zip › SD Figure 1/1C/HeLa ACE2 perm anti-Spike.tif]

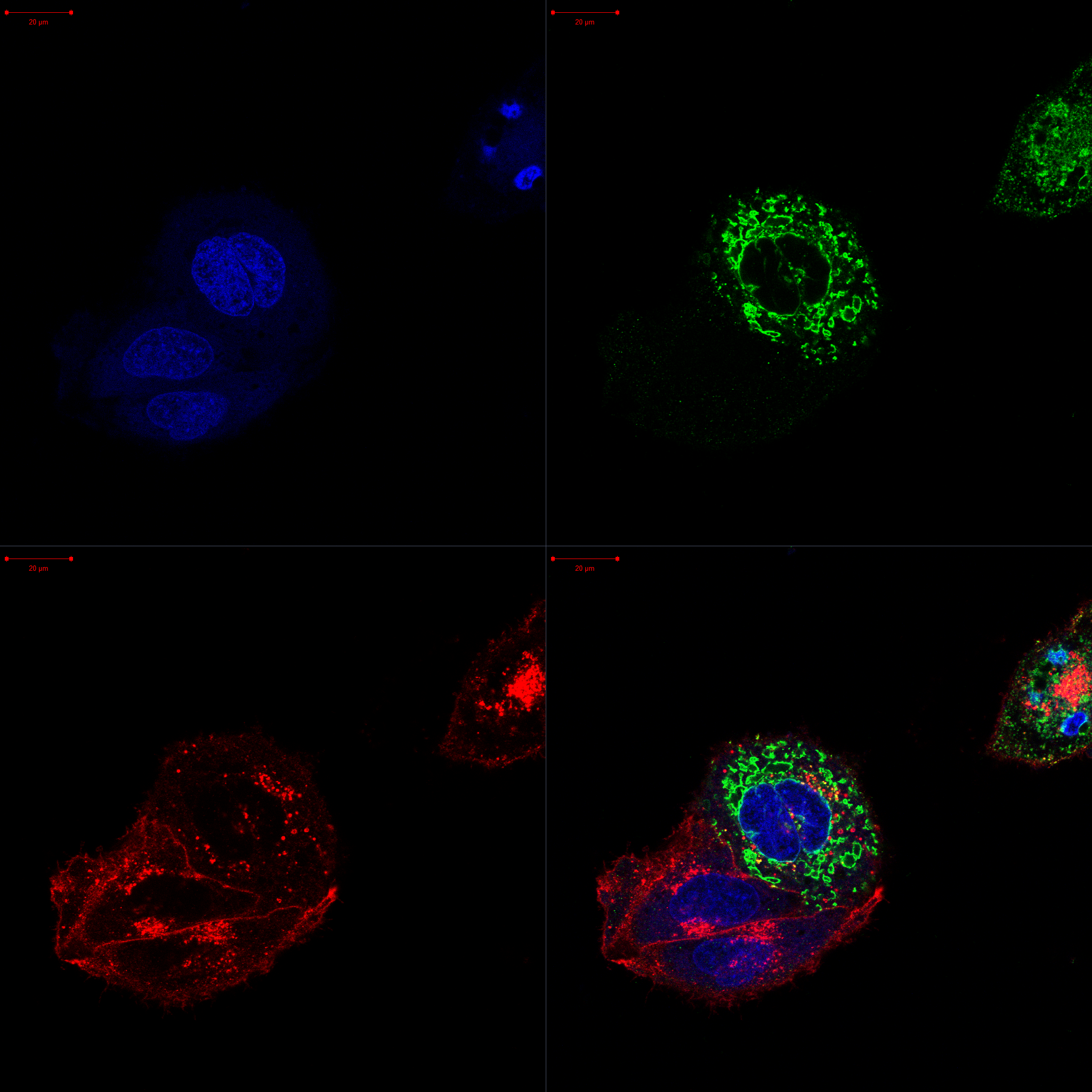

Supplement: Supplementary file 5 — Source Data for Figure 1 [file EMBR-24-e57224-s010.zip › SD Figure 1/1D/Figure 1D WT HeLa ACE2 perm anti-Spike anti-Bst2 c 2.tif]

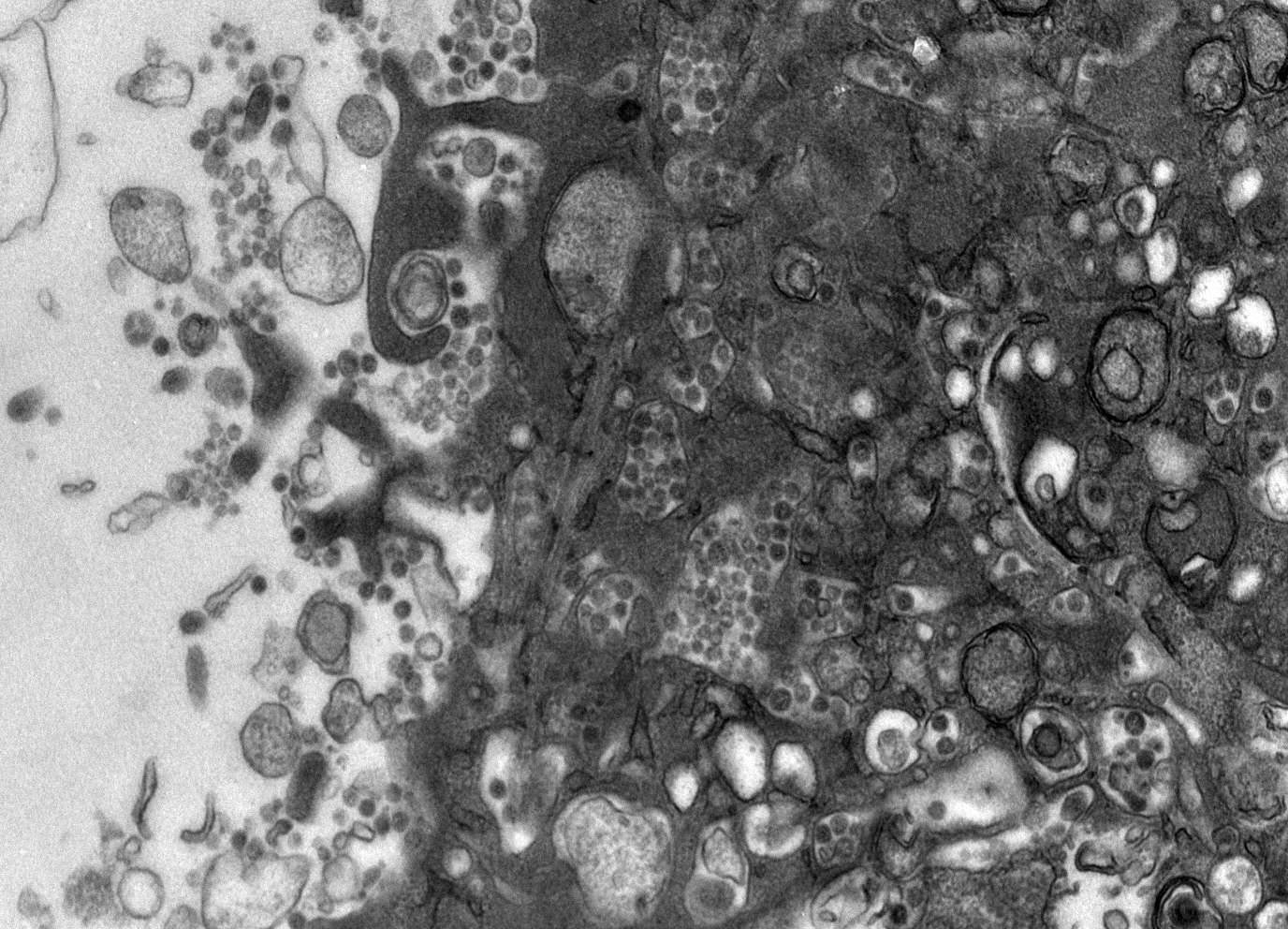

Supplement: Supplementary file 5 — Source Data for Figure 1 [file EMBR-24-e57224-s010.zip › SD Figure 1/1E/SD F2E part ii.jpg]

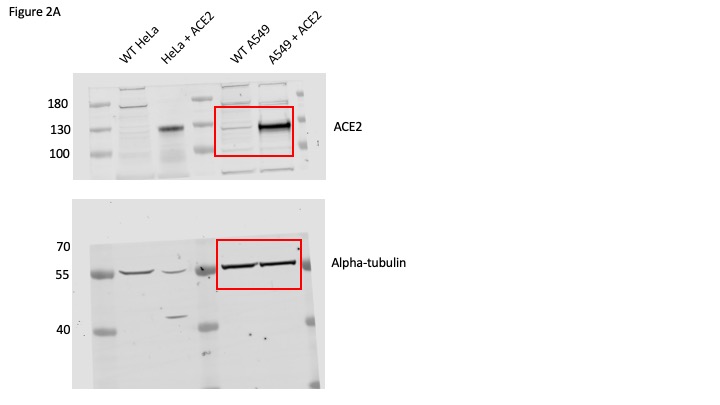

Supplement: Supplementary file 6 — Source Data for Figure 2 [file EMBR-24-e57224-s002.zip › SD Figure 2/2A/Figure 2A.jpeg]

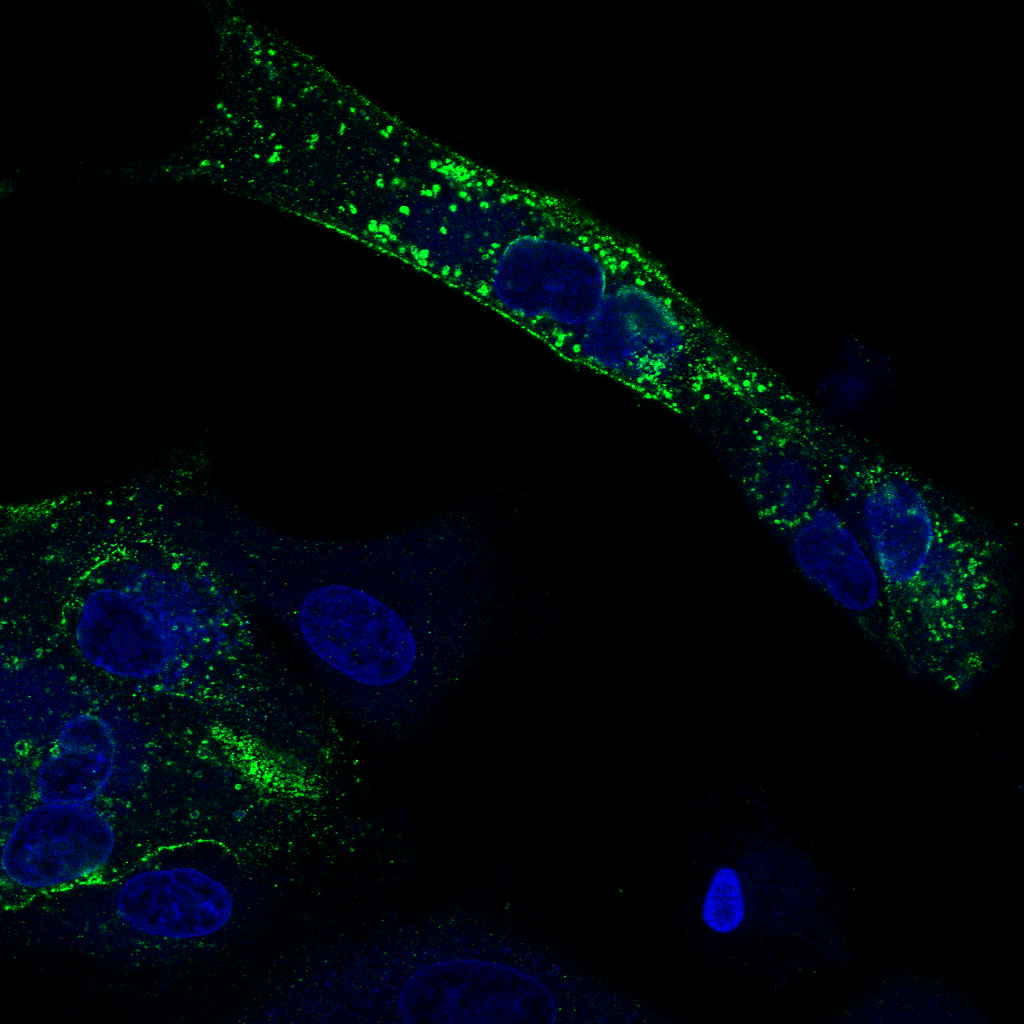

Supplement: Supplementary file 6 — Source Data for Figure 2 [file EMBR-24-e57224-s002.zip › SD Figure 2/2B/A549 WT ACE2 perm anti-Spike d.tif]

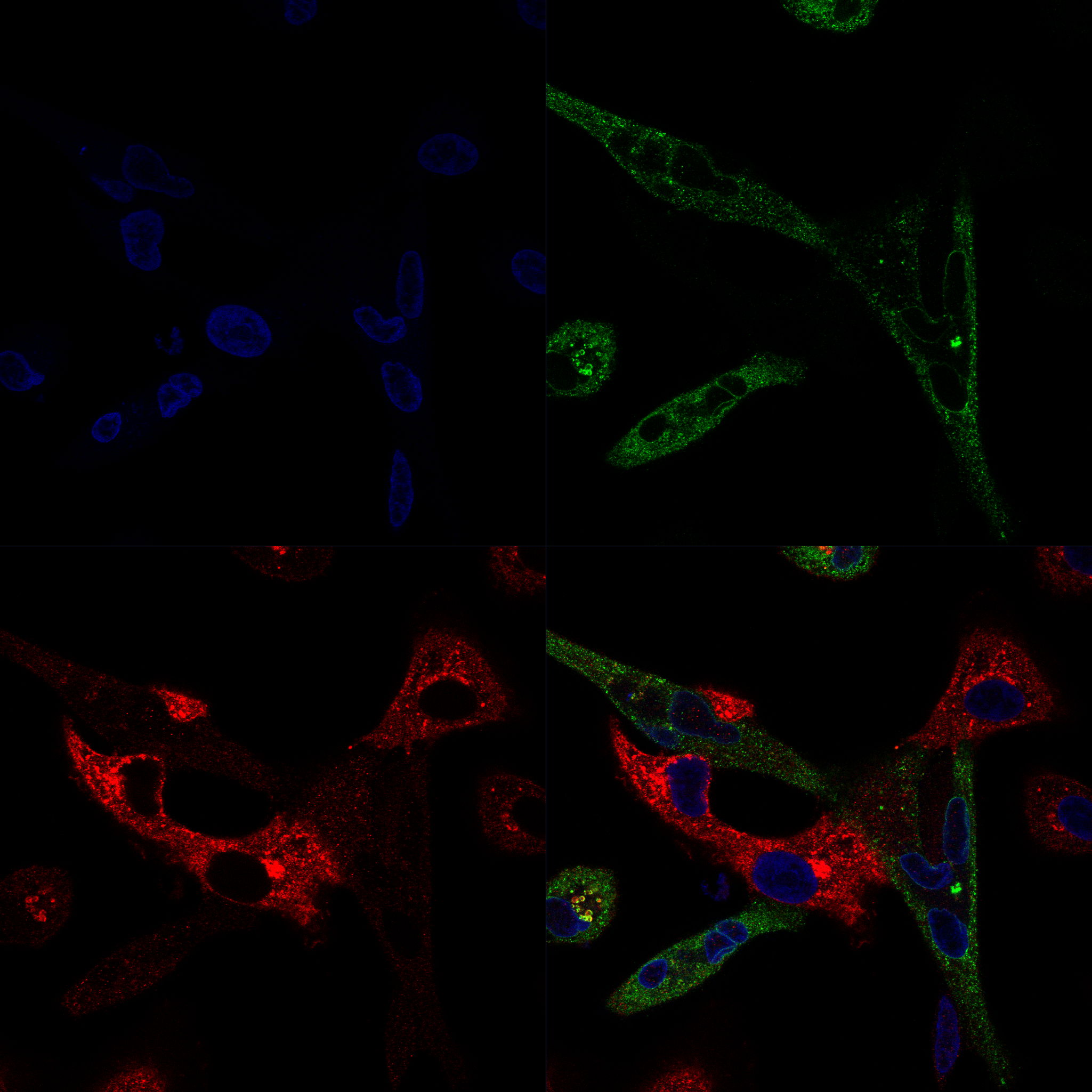

Supplement: Supplementary file 6 — Source Data for Figure 2 [file EMBR-24-e57224-s002.zip › SD Figure 2/2C/A549ACE2 IFN Spike Bst2 b.tif]

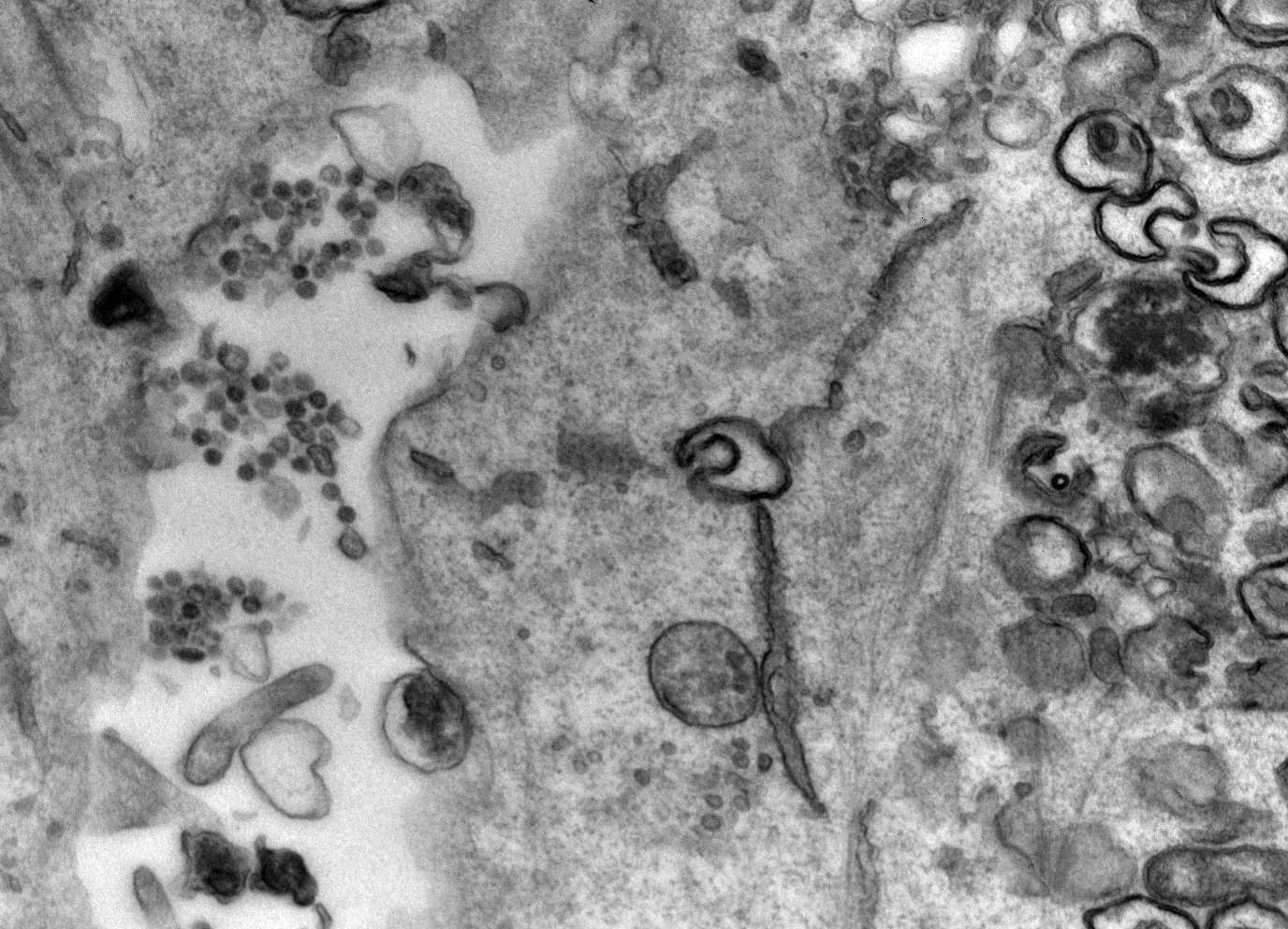

Supplement: Supplementary file 6 — Source Data for Figure 2 [file EMBR-24-e57224-s002.zip › SD Figure 2/2D/SD F2F part ii.jpg]

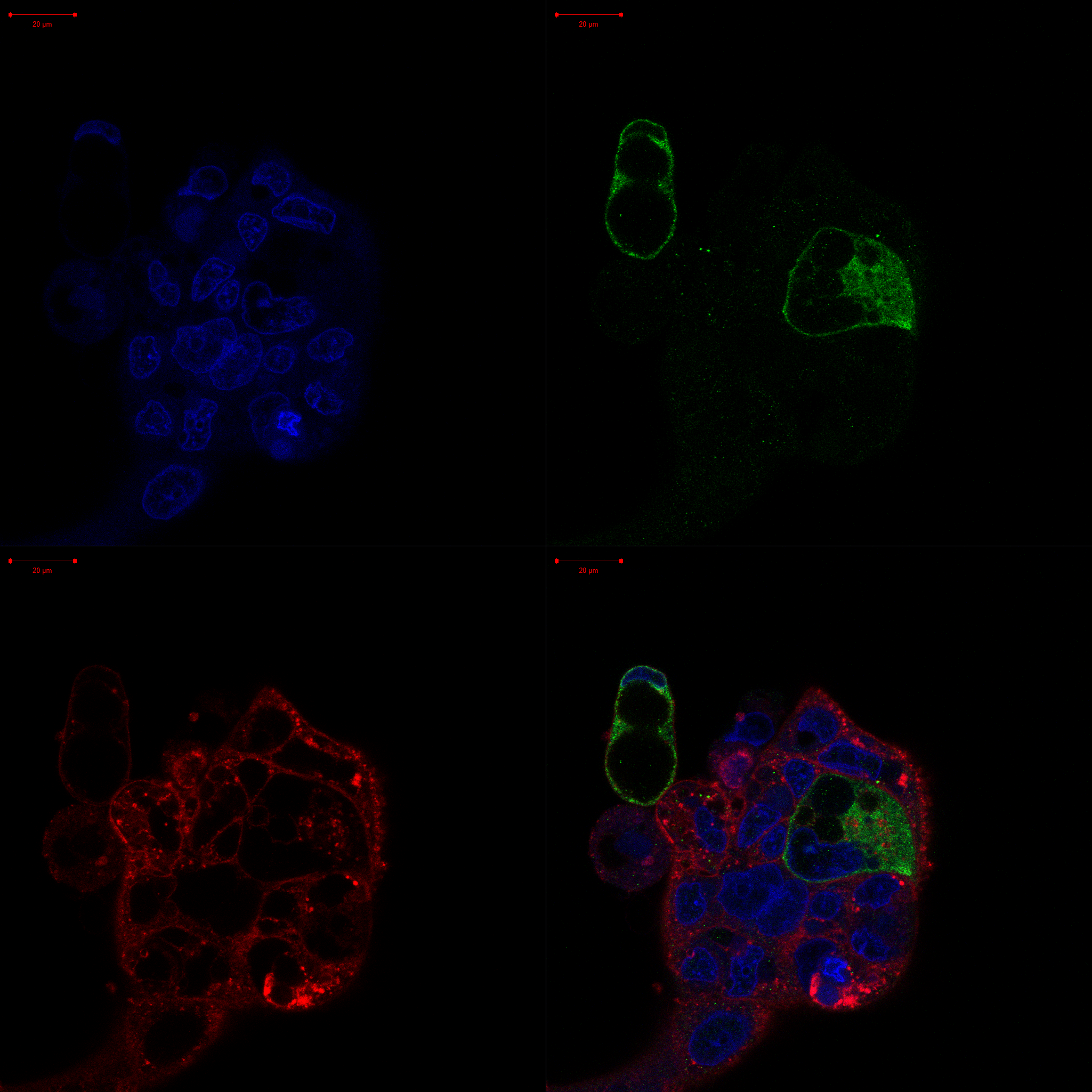

Supplement: Supplementary file 6 — Source Data for Figure 2 [file EMBR-24-e57224-s002.zip › SD Figure 2/2G/T84 perm anti-Spike anti-Bst2 b.tif]

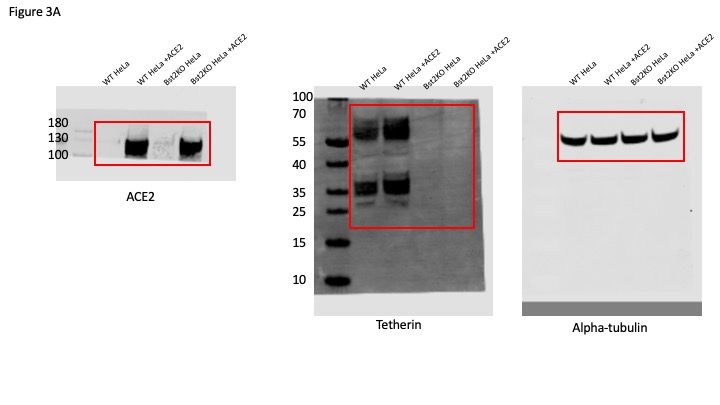

Supplement: Supplementary file 7 — Source Data for Figure 3 [file EMBR-24-e57224-s006.zip › SD Figure 3/3A/Figure 3A.jpeg]

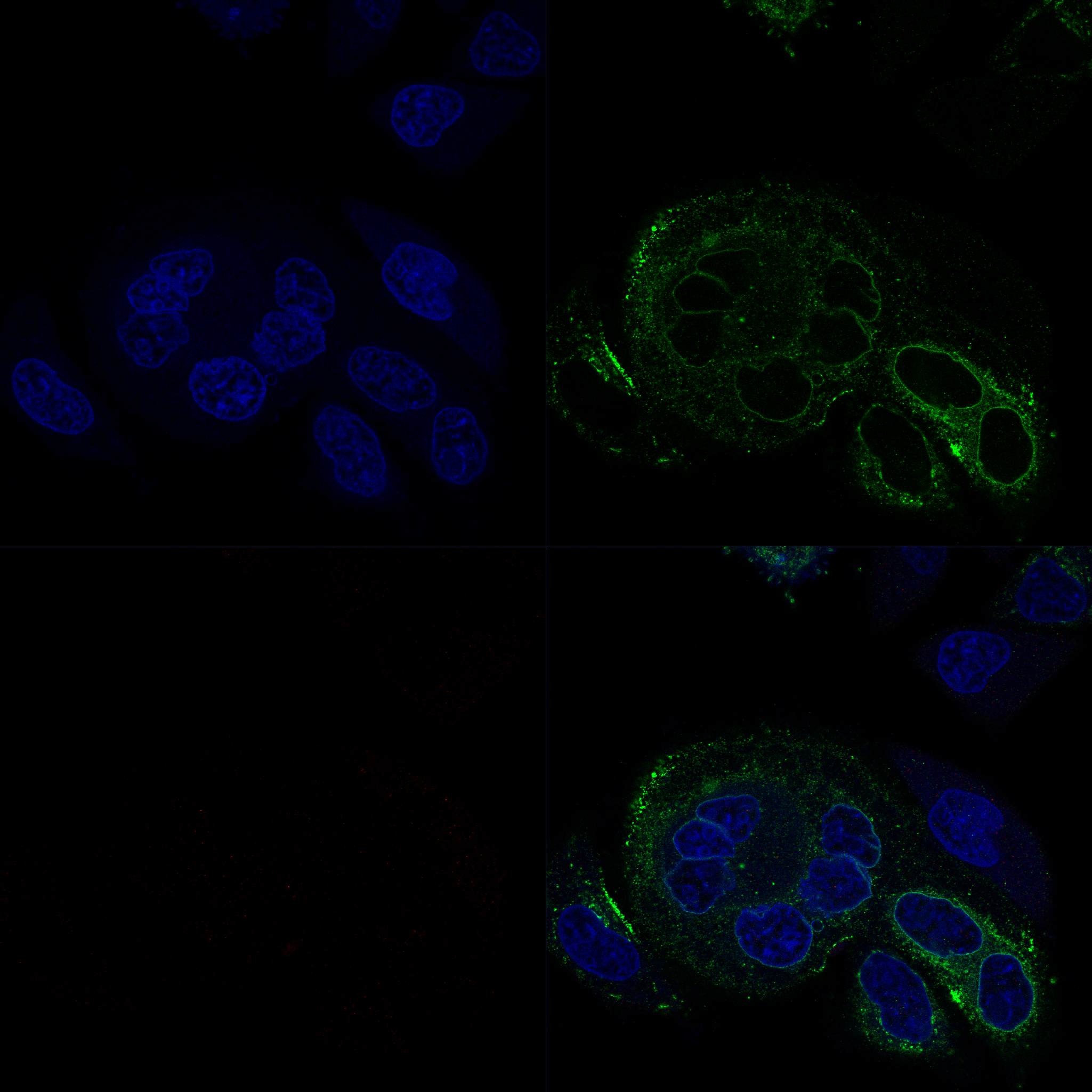

Supplement: Supplementary file 7 — Source Data for Figure 3 [file EMBR-24-e57224-s006.zip › SD Figure 3/3B/Bst2KO HeLa ACE2 perm anti-Spike anti-Bst2 b.tif]

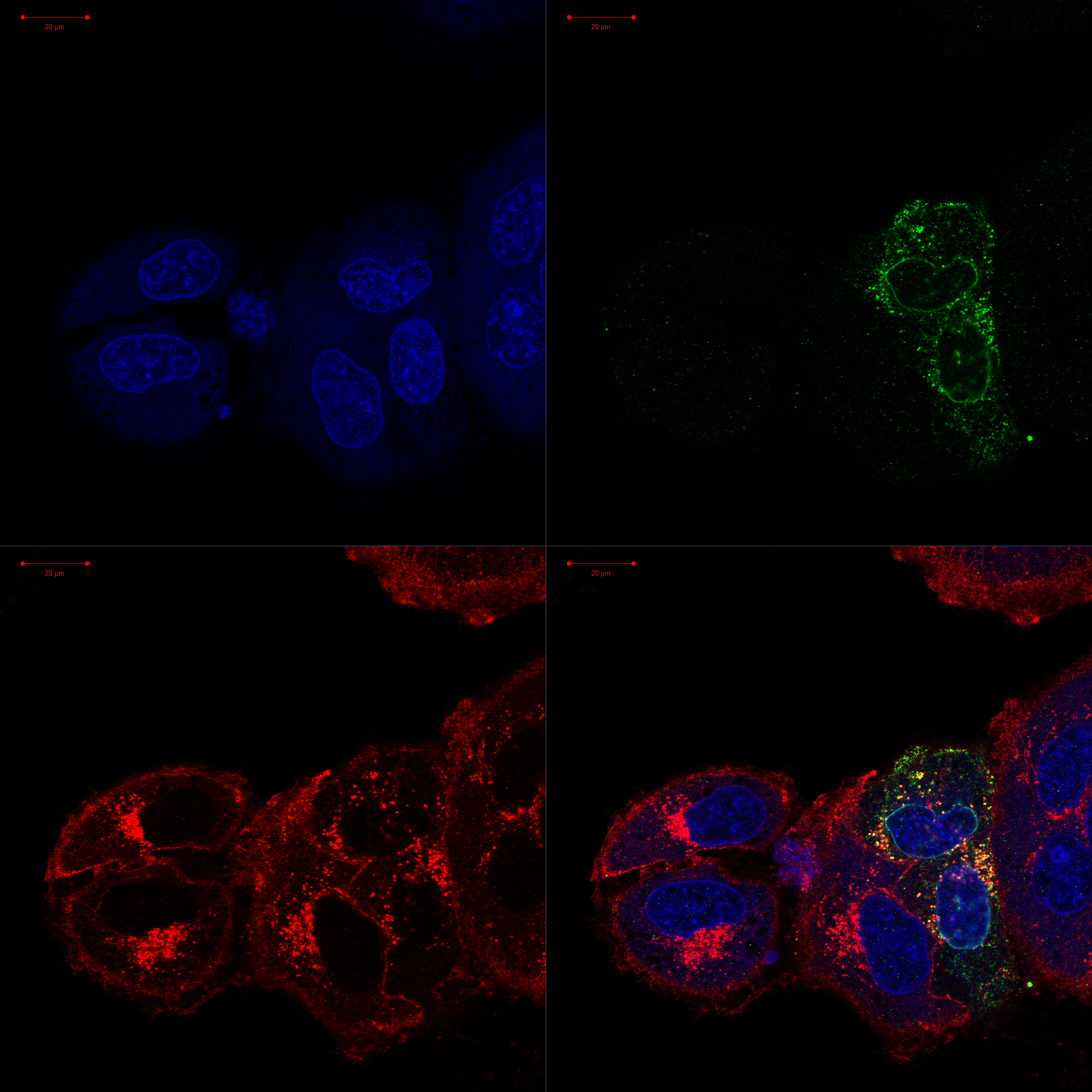

Supplement: Supplementary file 7 — Source Data for Figure 3 [file EMBR-24-e57224-s006.zip › SD Figure 3/3B/WT HeLa ACE2 perm anti-Spike anti-Bst2.tif]

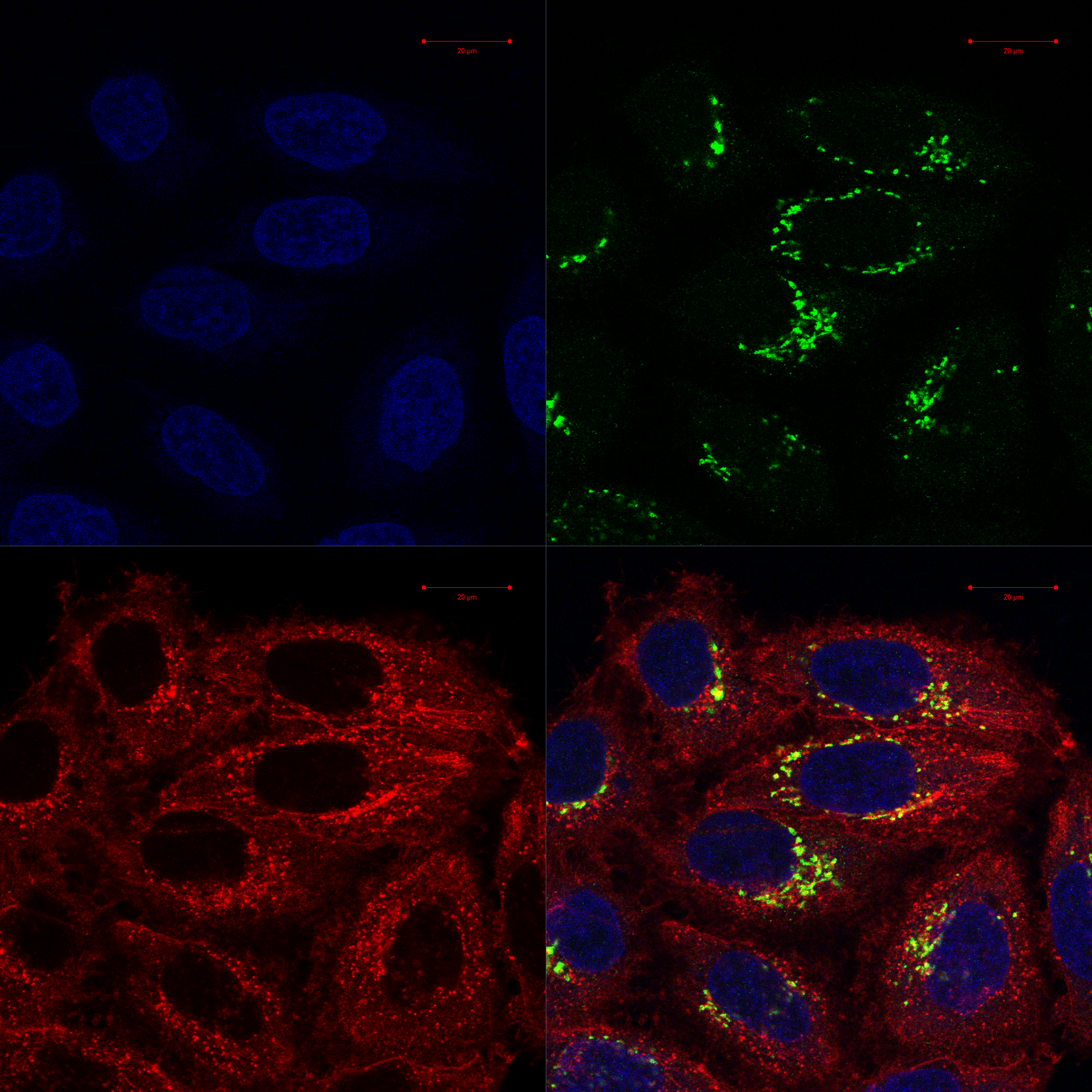

Supplement: Supplementary file 8 — Source Data for Figure 4 [file EMBR-24-e57224-s004.zip › SD Figure 4/4A/pQ CoV1 orf7a FLAG Bst2 3 SB.tif]

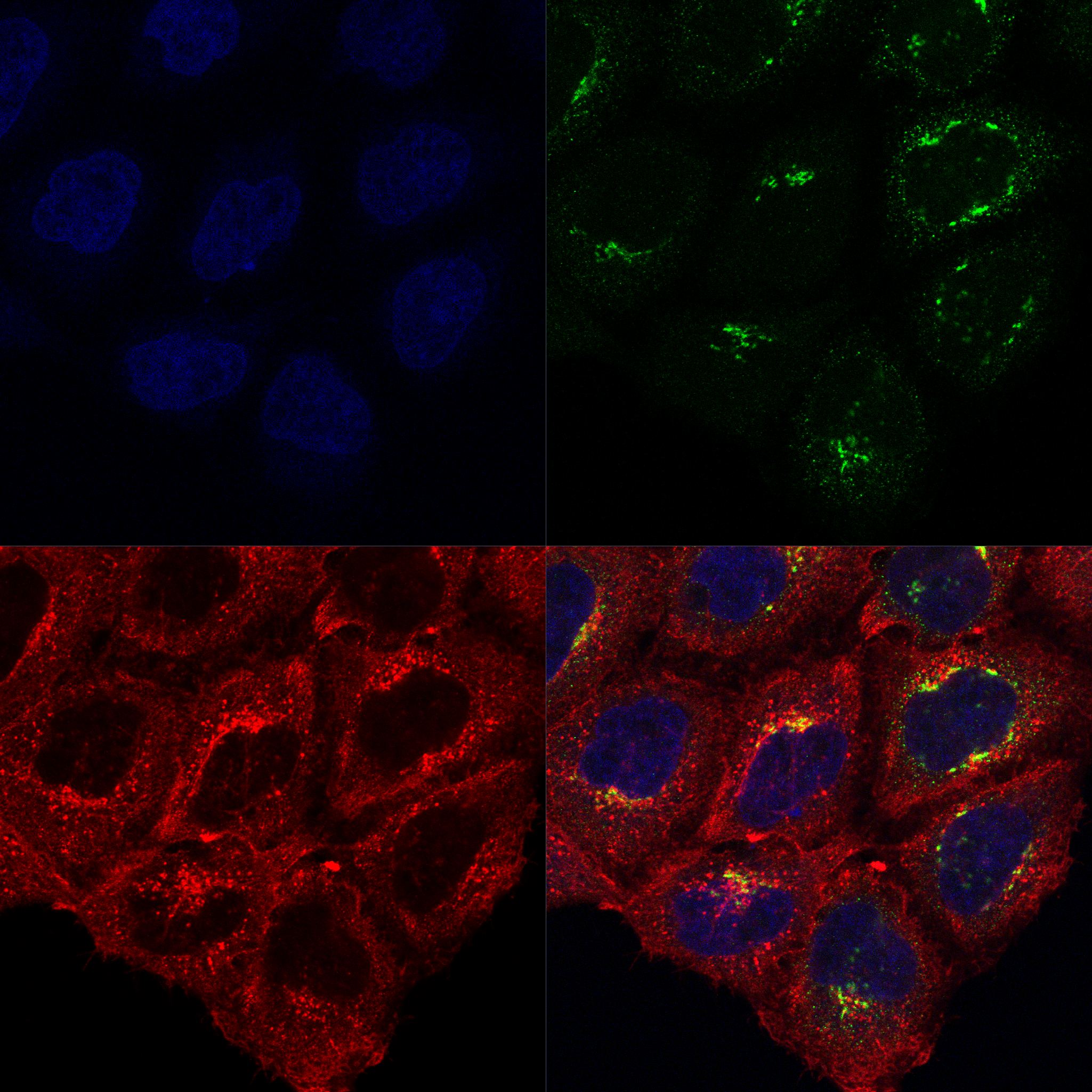

Supplement: Supplementary file 8 — Source Data for Figure 4 [file EMBR-24-e57224-s004.zip › SD Figure 4/4A/pQ CoV2 orf7a FLAG Bst2 2.tif]

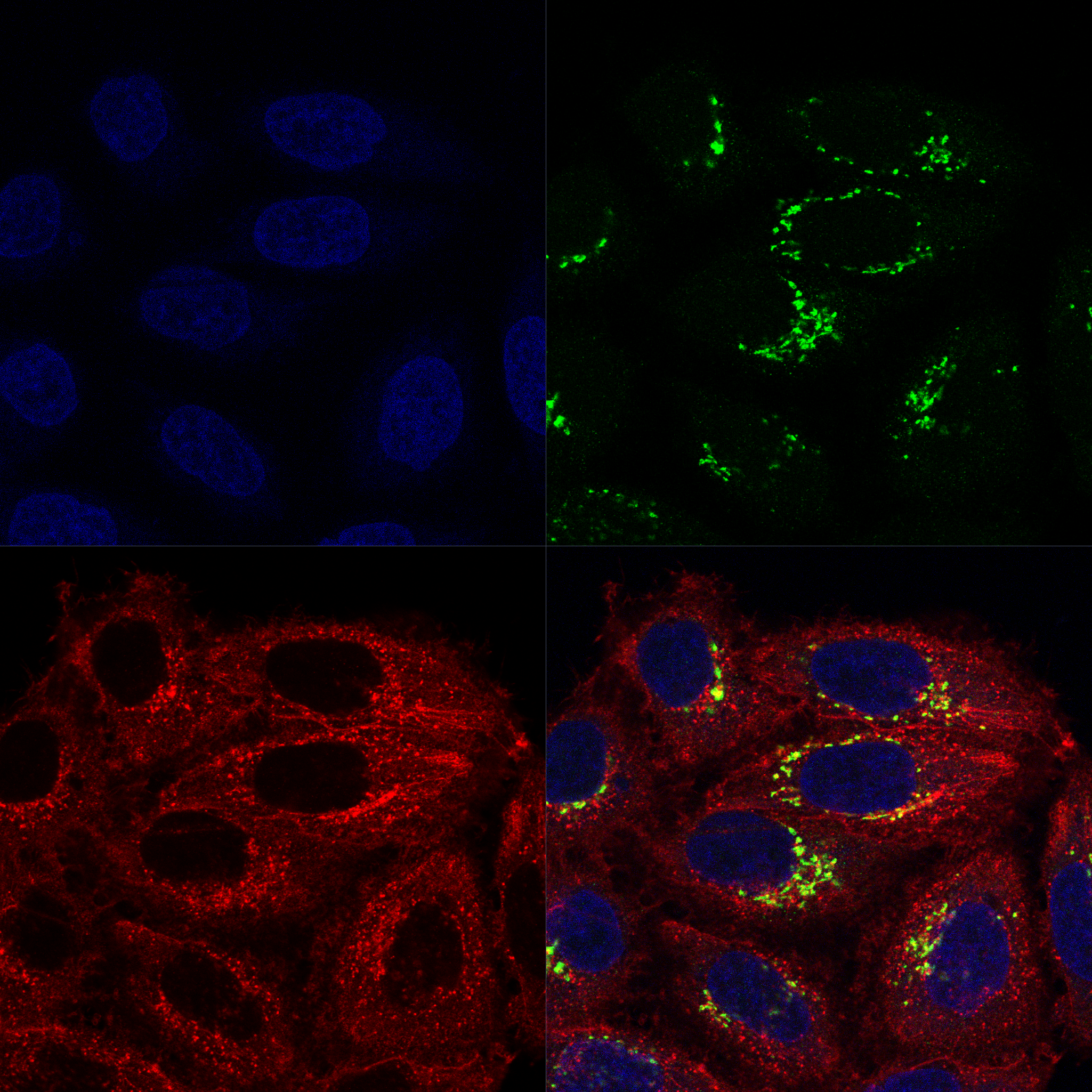

Supplement: Supplementary file 8 — Source Data for Figure 4 [file EMBR-24-e57224-s004.zip › SD Figure 4/4A/pQ CoV1 orf7a FLAG Bst2 3.tif]

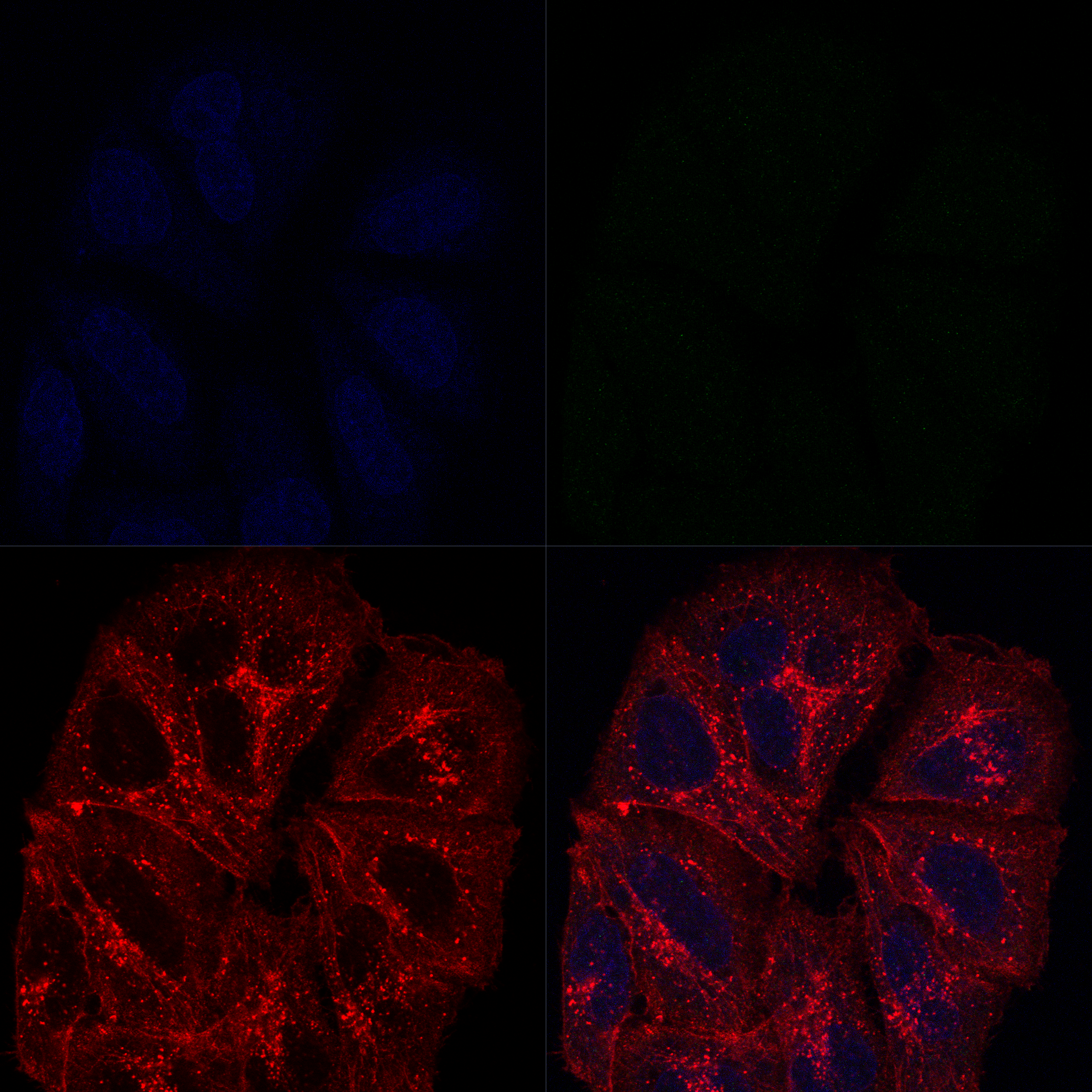

Supplement: Supplementary file 8 — Source Data for Figure 4 [file EMBR-24-e57224-s004.zip › SD Figure 4/4A/WT FLAG Bst2 3.tif]

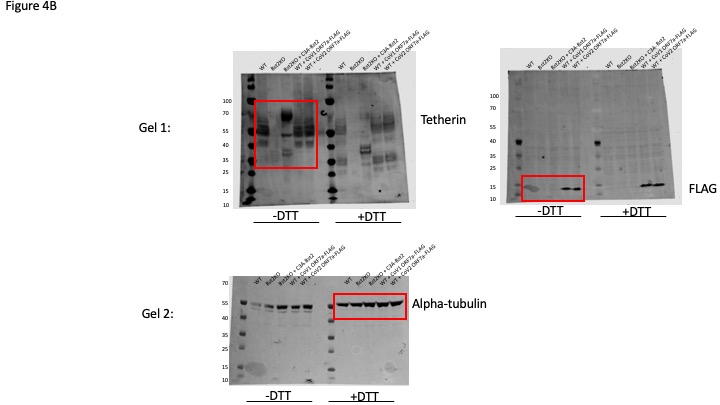

Supplement: Supplementary file 8 — Source Data for Figure 4 [file EMBR-24-e57224-s004.zip › SD Figure 4/4B/Figure 4B.jpeg]

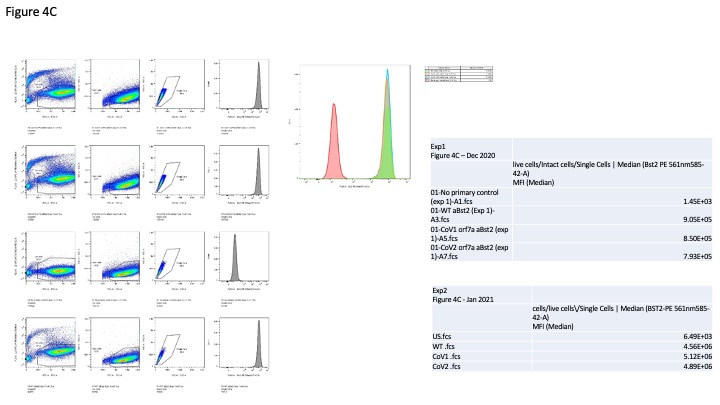

Supplement: Supplementary file 8 — Source Data for Figure 4 [file EMBR-24-e57224-s004.zip › SD Figure 4/4C/Figure 4C.jpeg]

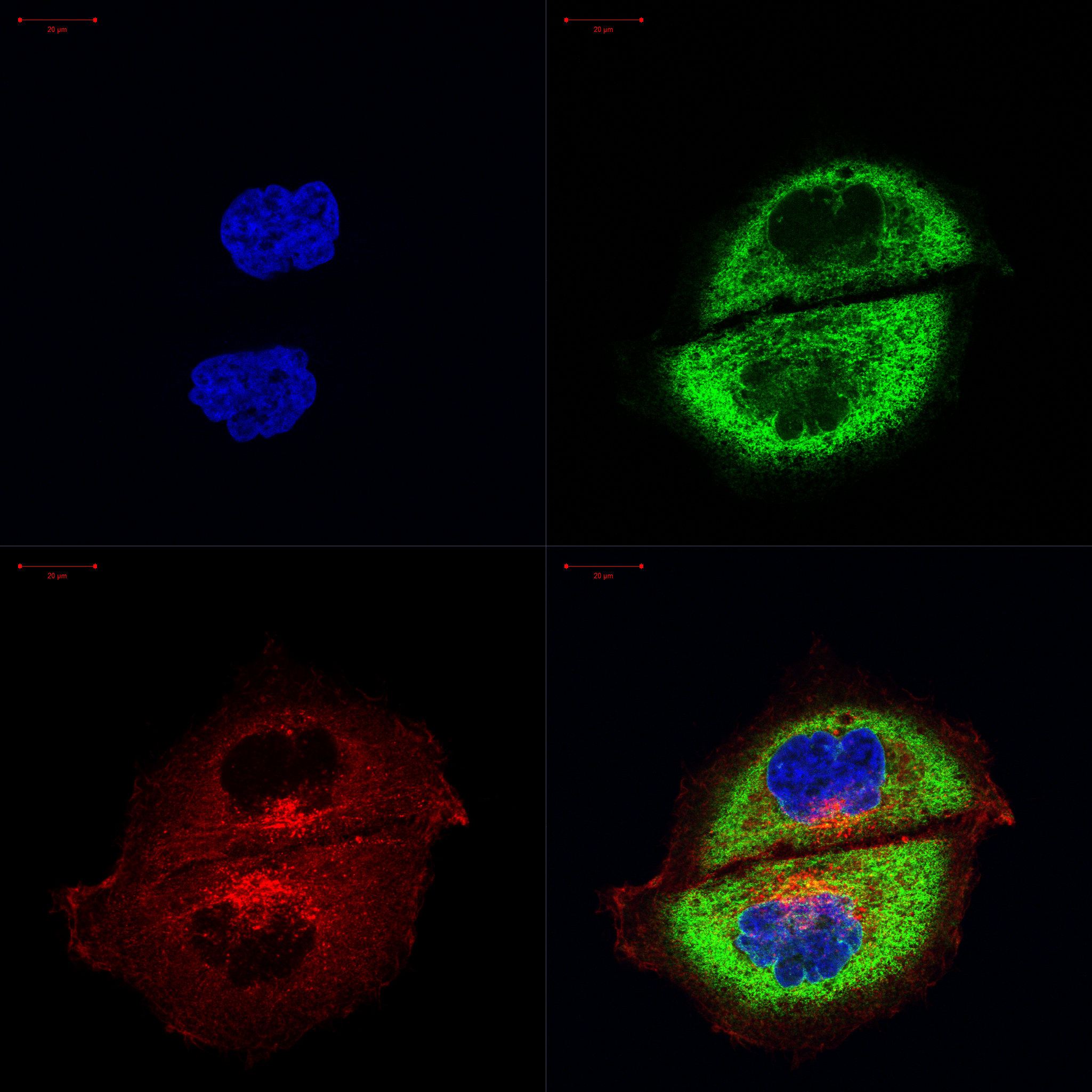

Supplement: Supplementary file 8 — Source Data for Figure 4 [file EMBR-24-e57224-s004.zip › SD Figure 4/4D/ss-HA-Spike WITH Dox anti-HA anti-bst2 c.tif]

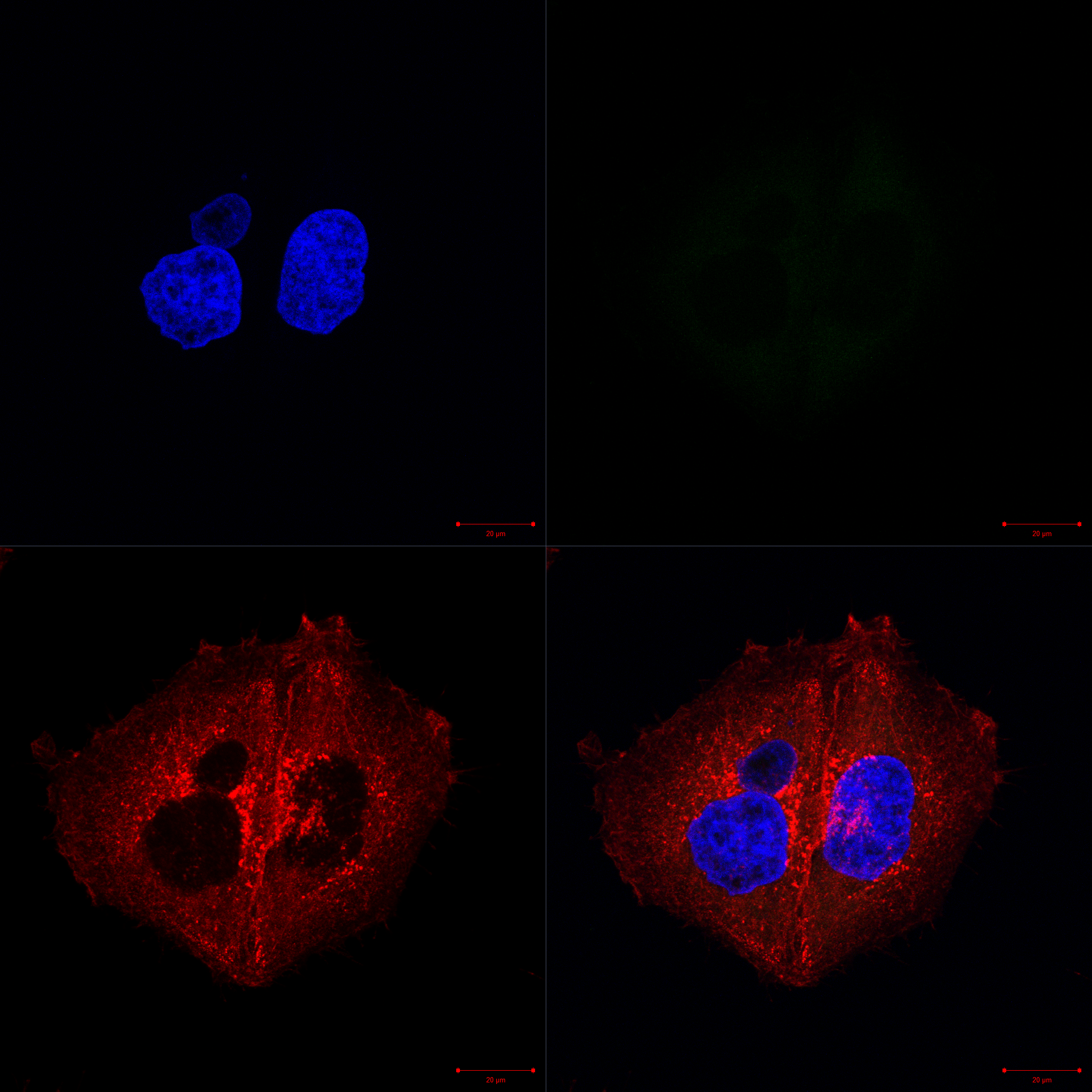

Supplement: Supplementary file 8 — Source Data for Figure 4 [file EMBR-24-e57224-s004.zip › SD Figure 4/4D/ss-HA-Spike NO Dox anti-HA anti-bst2 c.tif]

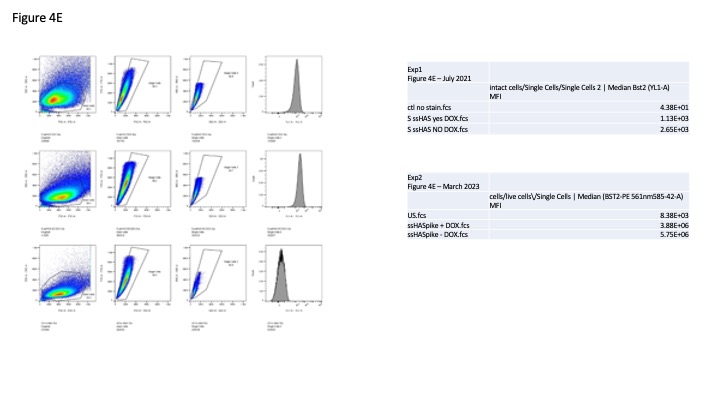

Supplement: Supplementary file 8 — Source Data for Figure 4 [file EMBR-24-e57224-s004.zip › SD Figure 4/4E/Figure 4E.jpeg]

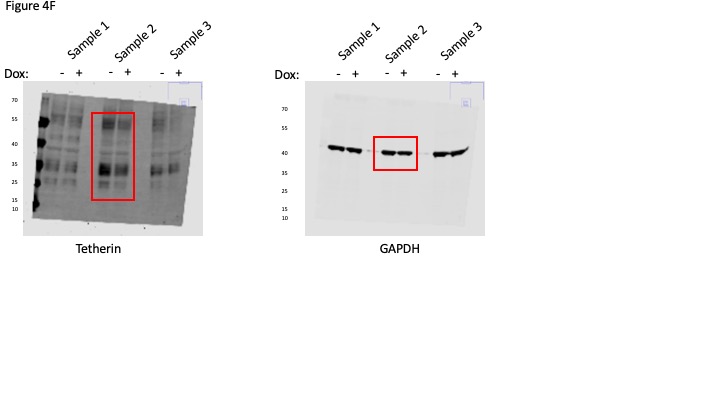

Supplement: Supplementary file 8 — Source Data for Figure 4 [file EMBR-24-e57224-s004.zip › SD Figure 4/4F/Figure 4F.jpeg]

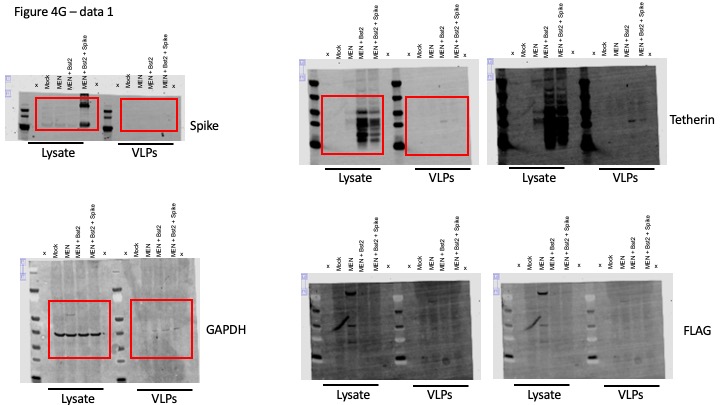

Supplement: Supplementary file 8 — Source Data for Figure 4 [file EMBR-24-e57224-s004.zip › SD Figure 4/4G/Figure 4G Part A.jpeg]

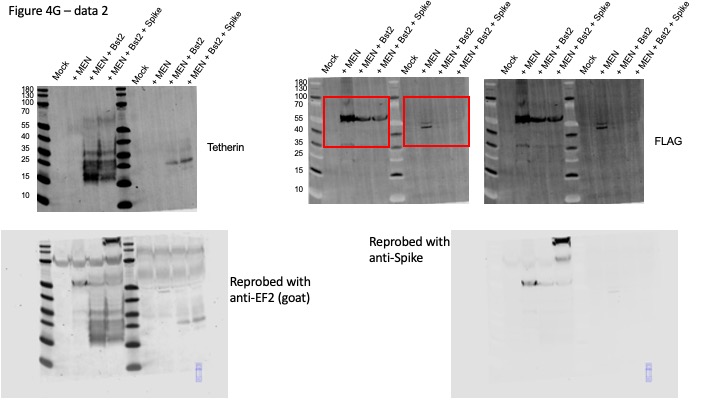

Supplement: Supplementary file 8 — Source Data for Figure 4 [file EMBR-24-e57224-s004.zip › SD Figure 4/4G/Figure 4G Part B.jpeg]

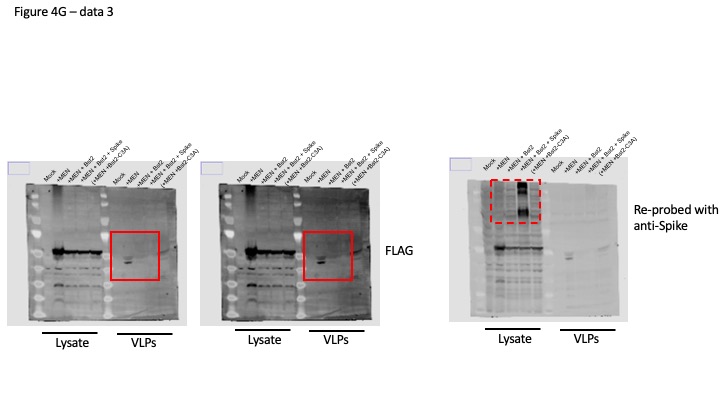

Supplement: Supplementary file 8 — Source Data for Figure 4 [file EMBR-24-e57224-s004.zip › SD Figure 4/4G/Figure 4G Part C.jpeg]

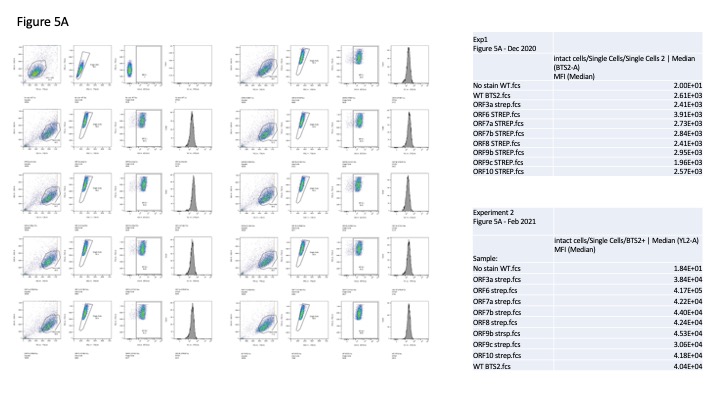

Supplement: Supplementary file 9 — Source Data for Figure 5 [file EMBR-24-e57224-s008.zip › SD Figure 5/5A/Figure 5A.jpeg]

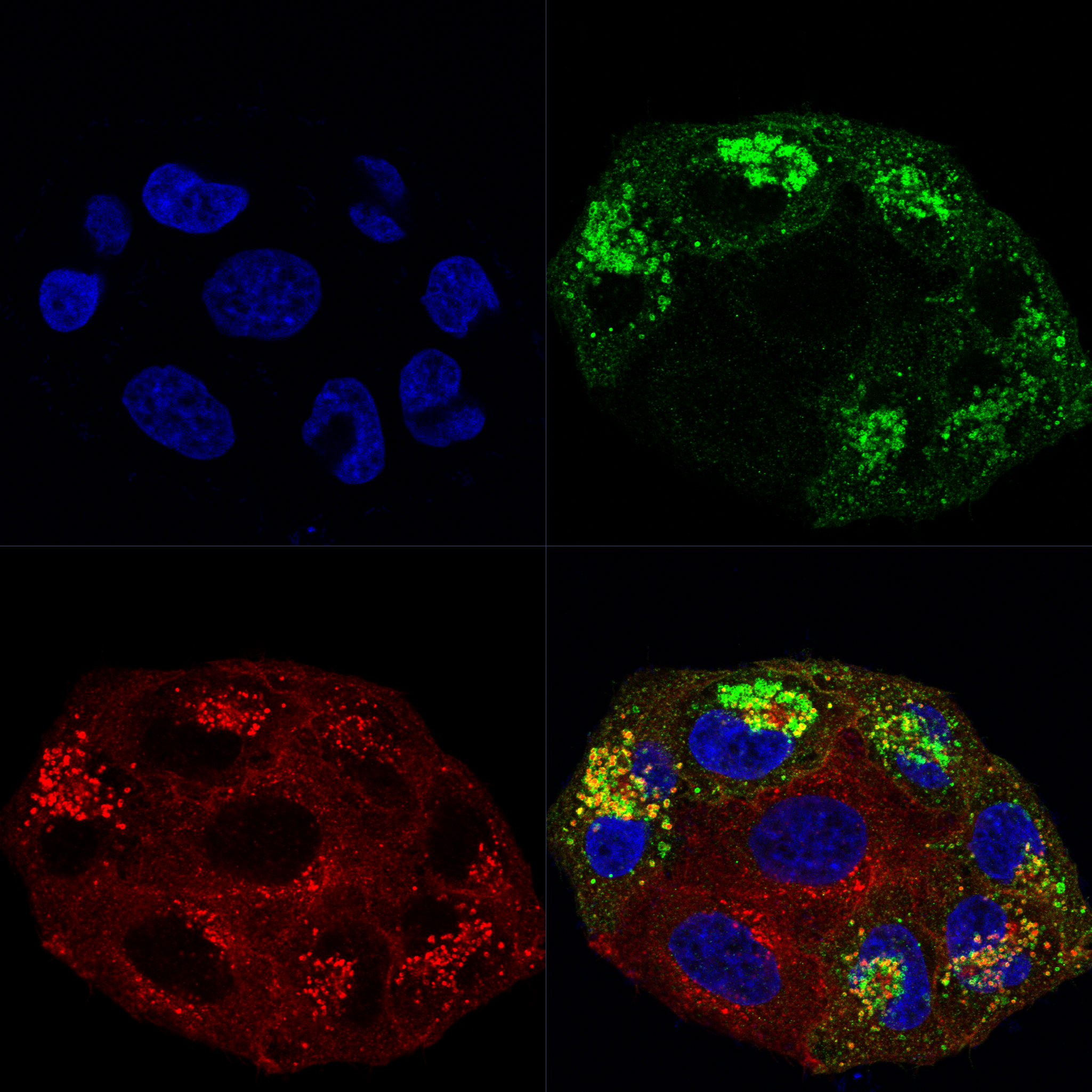

Supplement: Supplementary file 9 — Source Data for Figure 5 [file EMBR-24-e57224-s008.zip › SD Figure 5/5B/orf3a anti-strep anti-bst2 c zoom.tif]

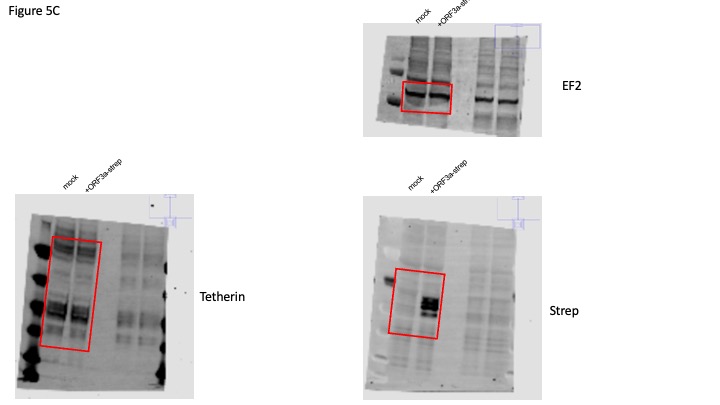

Supplement: Supplementary file 9 — Source Data for Figure 5 [file EMBR-24-e57224-s008.zip › SD Figure 5/5C/Figure 5C.jpeg]

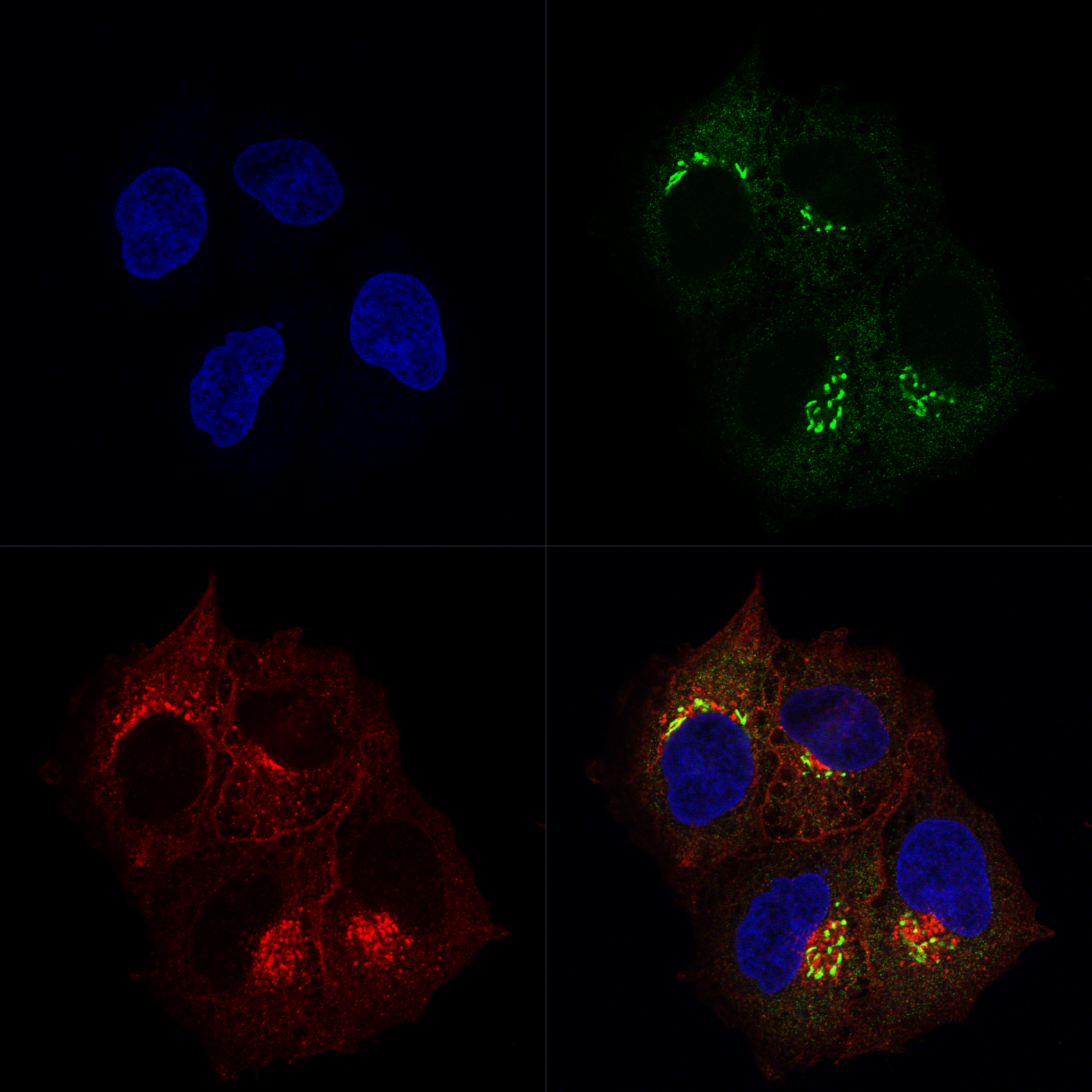

Supplement: Supplementary file 9 — Source Data for Figure 5 [file EMBR-24-e57224-s008.zip › SD Figure 5/5D/mock TGN46 Bst2 72hr adj.tif]

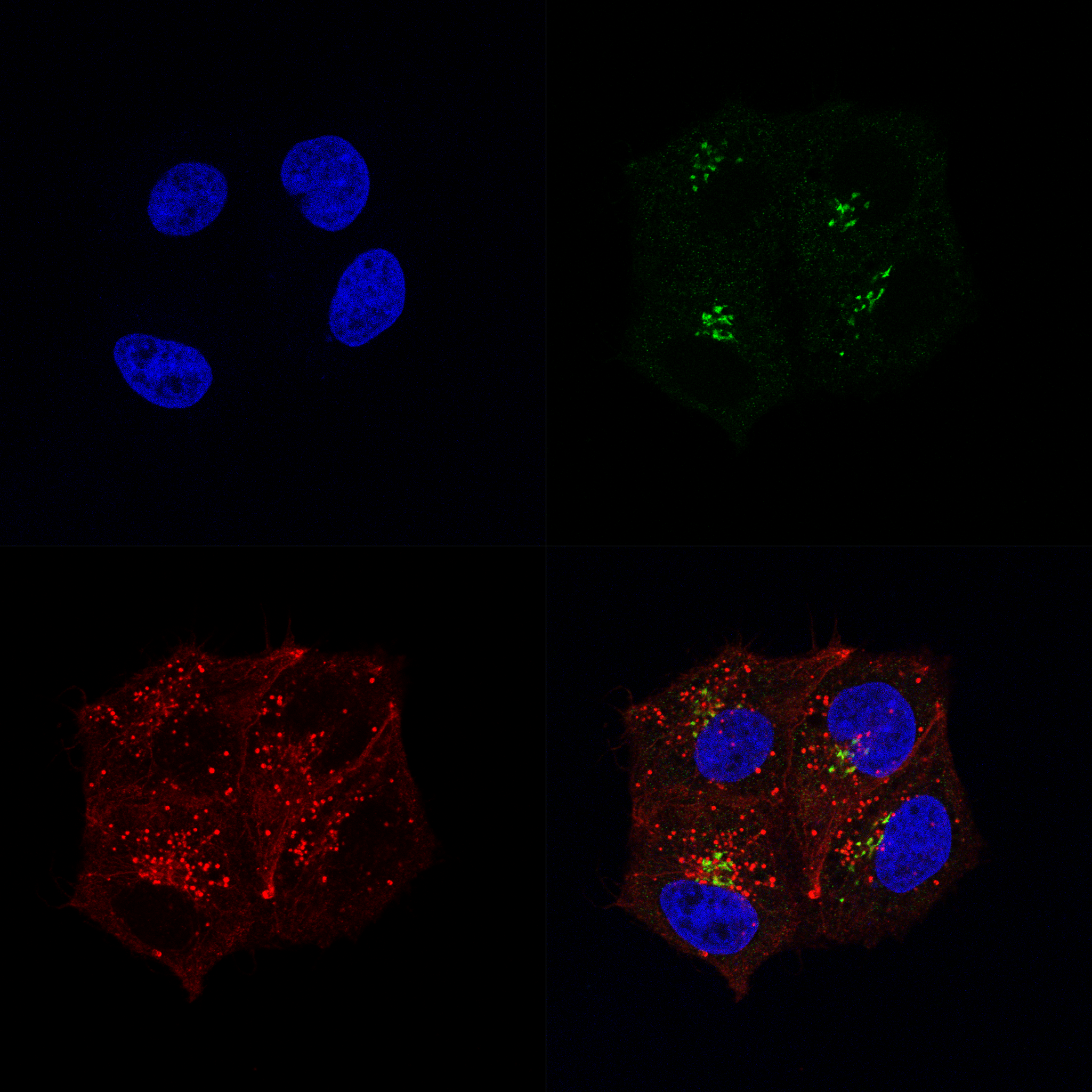

Supplement: Supplementary file 9 — Source Data for Figure 5 [file EMBR-24-e57224-s008.zip › SD Figure 5/5D/ORF3a TGN46 Bst2 72hr e adj.tif]

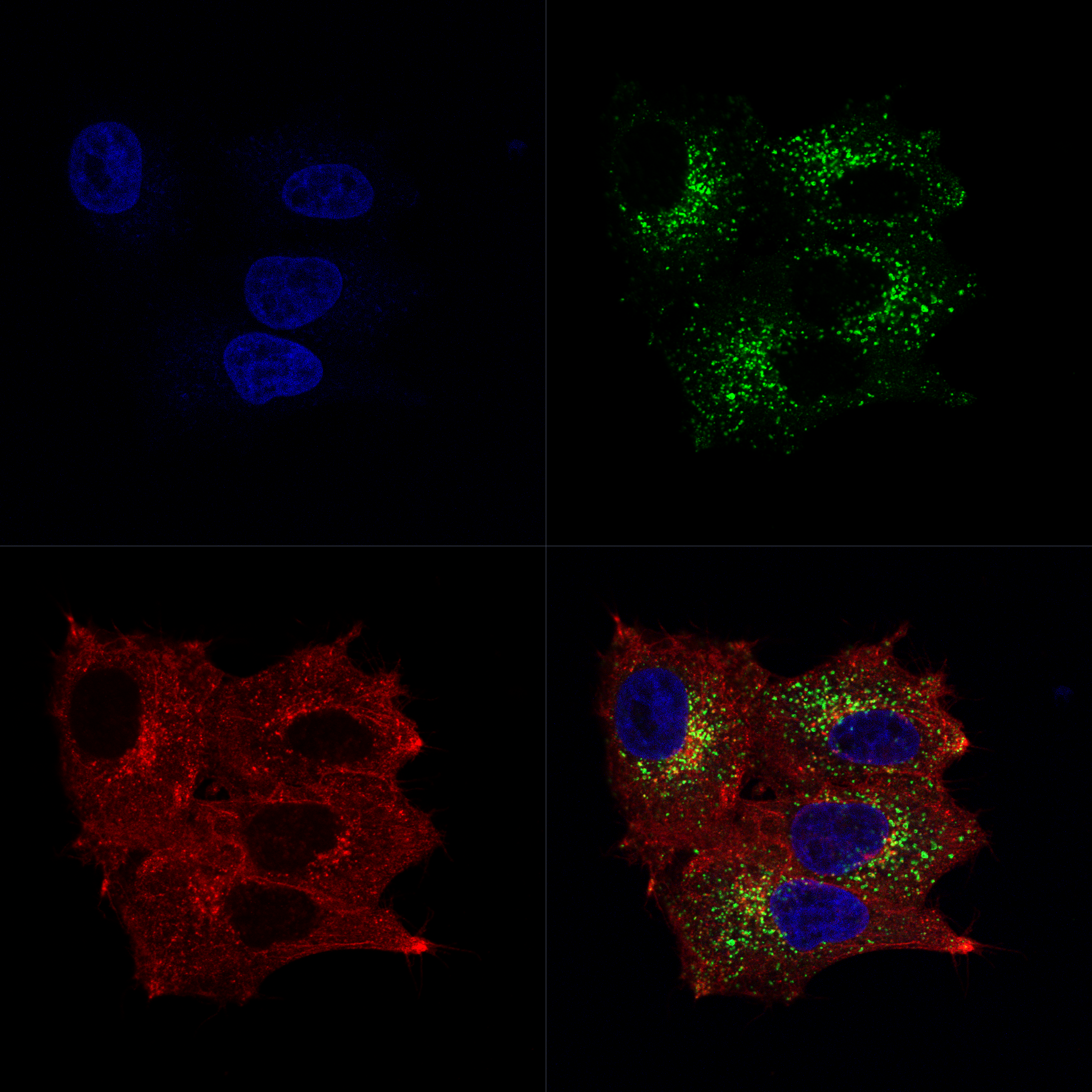

Supplement: Supplementary file 9 — Source Data for Figure 5 [file EMBR-24-e57224-s008.zip › SD Figure 5/5F/mock LAMP1Bst2 72hr e.tif]

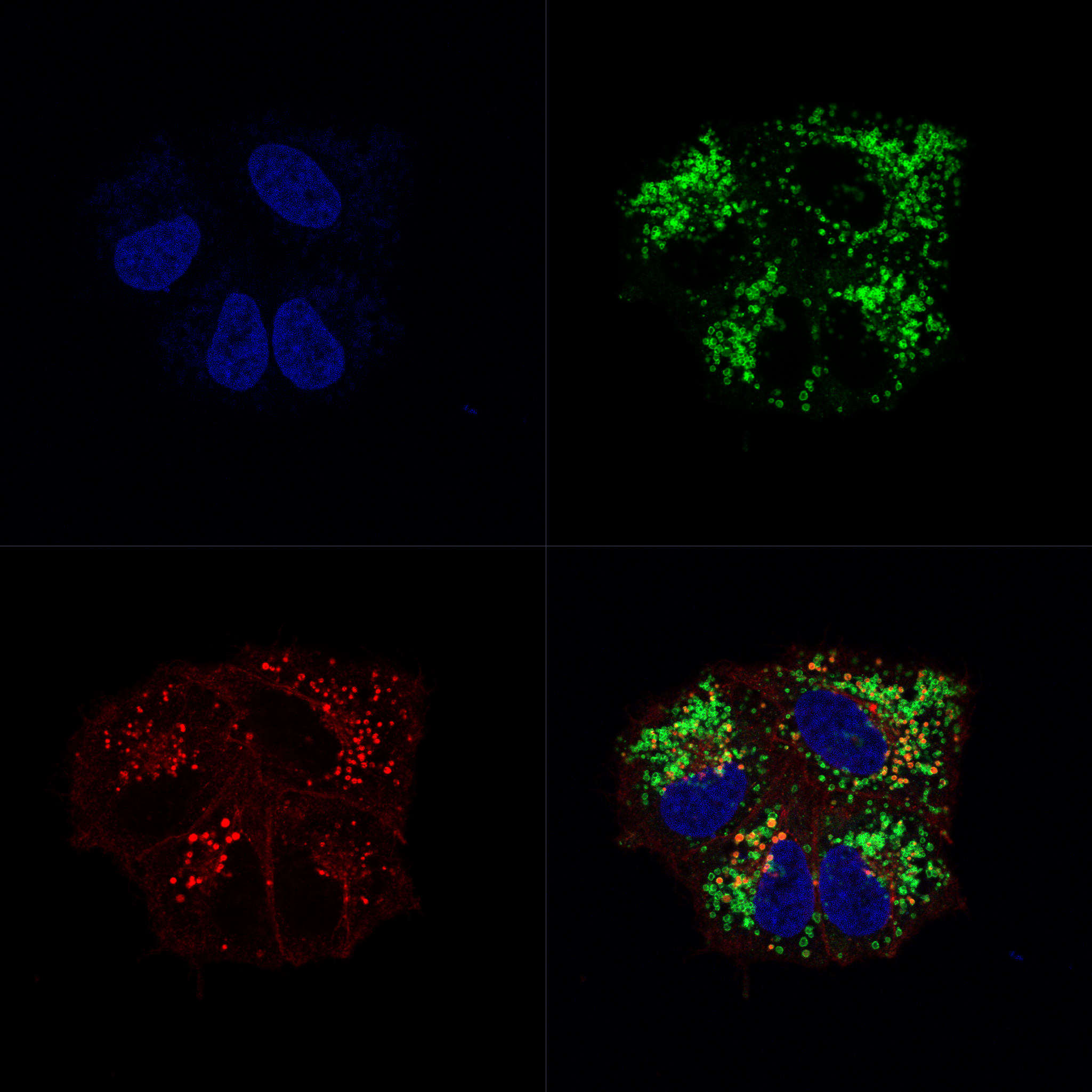

Supplement: Supplementary file 9 — Source Data for Figure 5 [file EMBR-24-e57224-s008.zip › SD Figure 5/5F/ORF3aLAMP1Bst2 72hr b.tif]

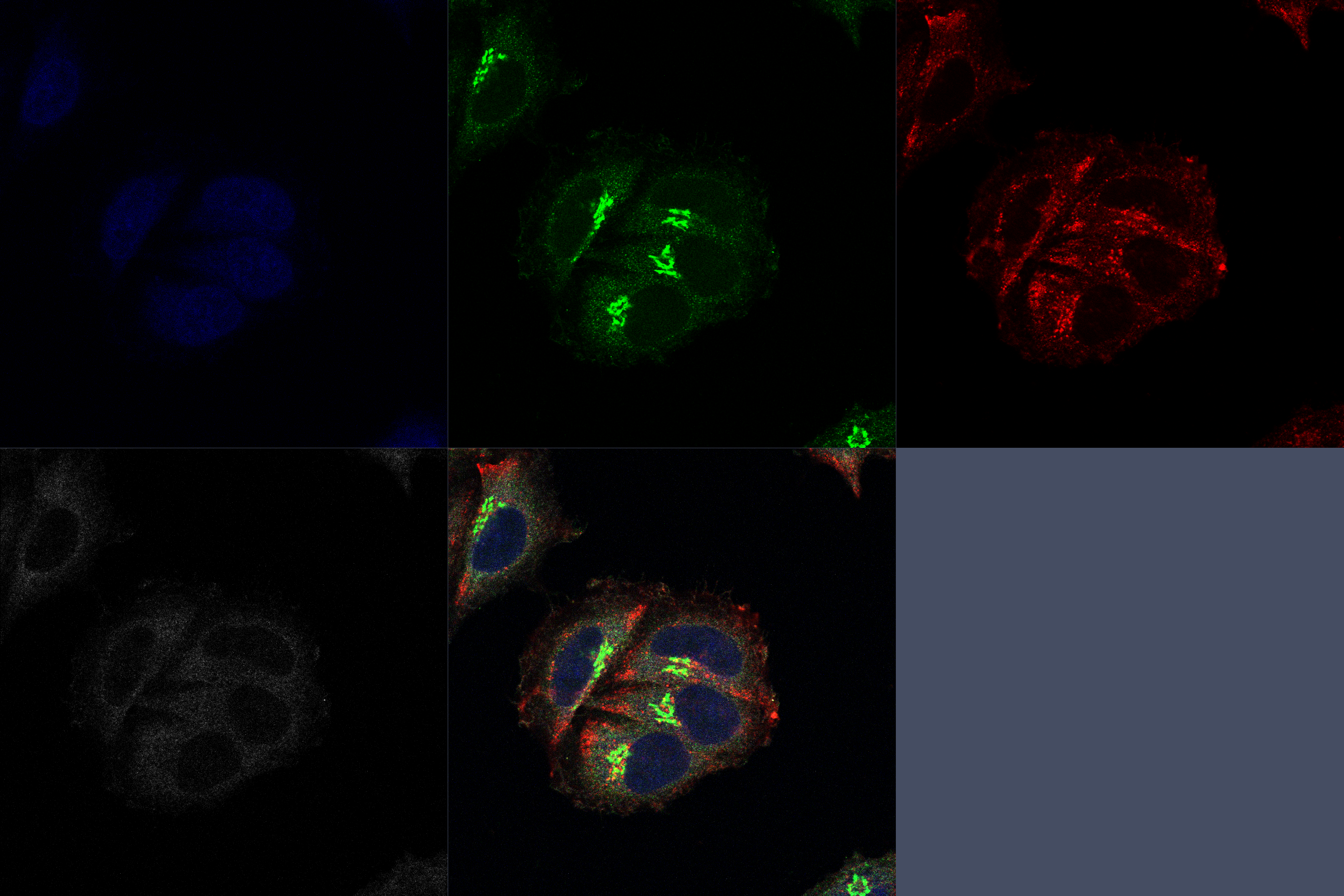

Supplement: Supplementary file 9 — Source Data for Figure 5 [file EMBR-24-e57224-s008.zip › SD Figure 5/5H/Bst2 fed anti-TGN46 anti-rb anti-strep MOCK b.tif]

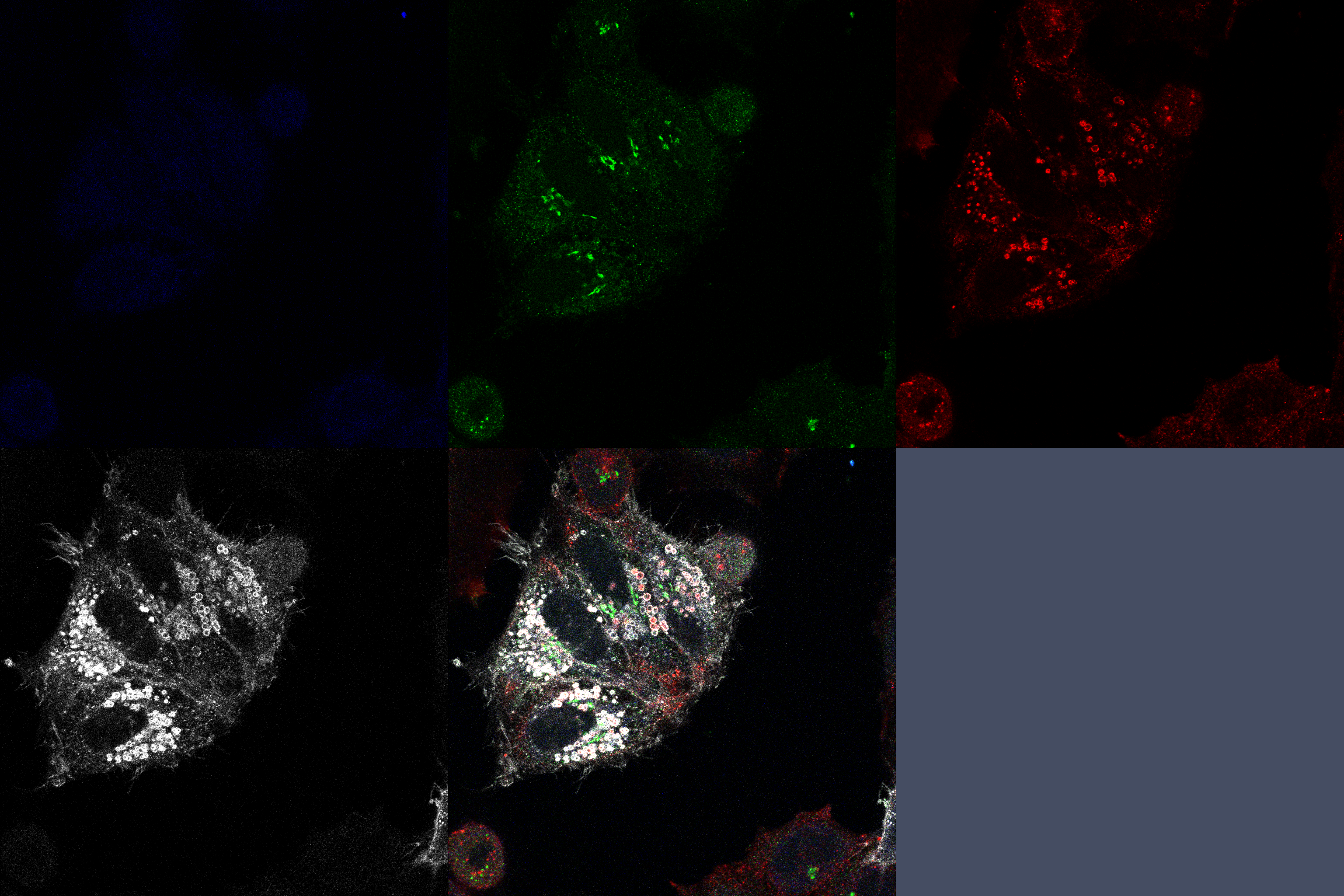

Supplement: Supplementary file 9 — Source Data for Figure 5 [file EMBR-24-e57224-s008.zip › SD Figure 5/5H/Bst2 fed anti-TGN46 anti-rb anti-strep e.tif]

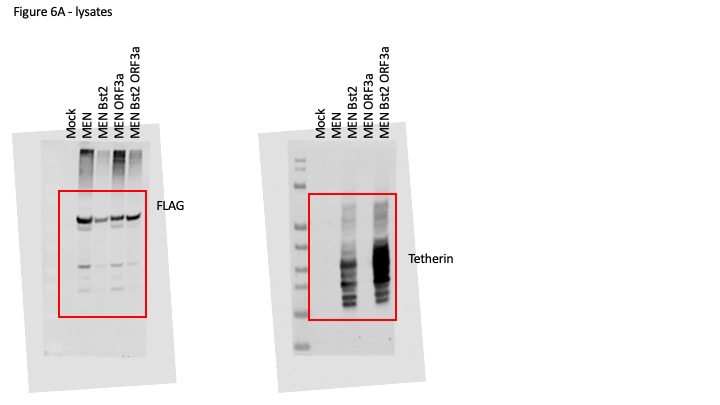

Supplement: Supplementary file 10 — Source Data for Figure 6 [file EMBR-24-e57224-s007.zip › SD Figure 6/6A/Figure 6A Part A.jpeg]

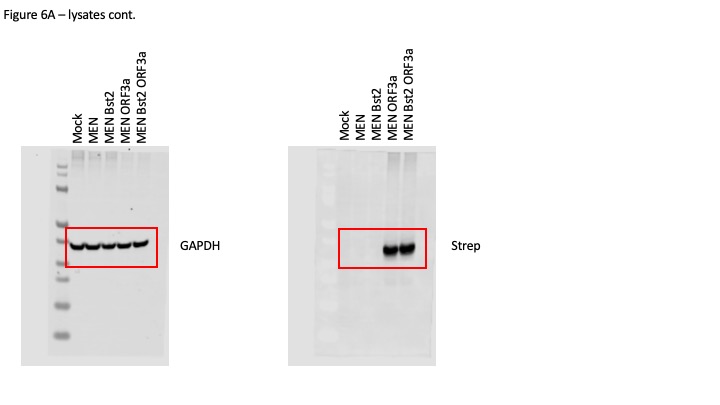

Supplement: Supplementary file 10 — Source Data for Figure 6 [file EMBR-24-e57224-s007.zip › SD Figure 6/6A/Figure 6A Part B.jpeg]

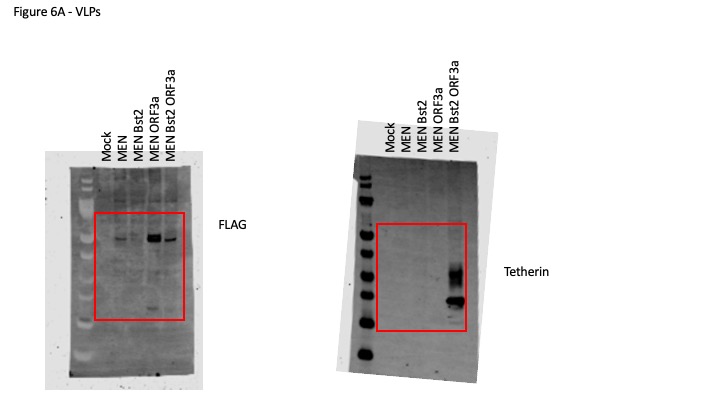

Supplement: Supplementary file 10 — Source Data for Figure 6 [file EMBR-24-e57224-s007.zip › SD Figure 6/6A/Figure 6A Part C.jpeg]

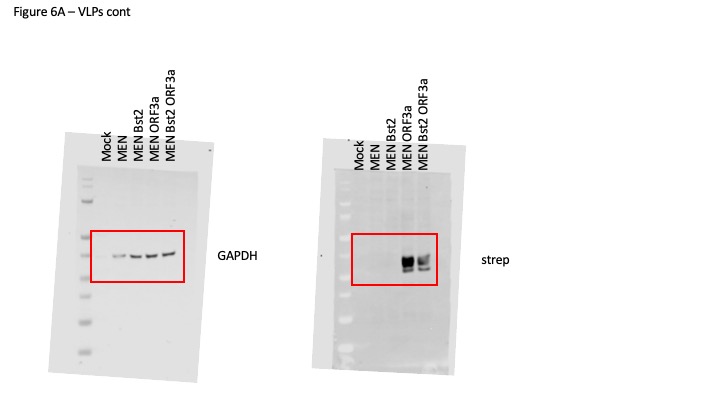

Supplement: Supplementary file 10 — Source Data for Figure 6 [file EMBR-24-e57224-s007.zip › SD Figure 6/6A/Figure 6A Part D.jpeg]

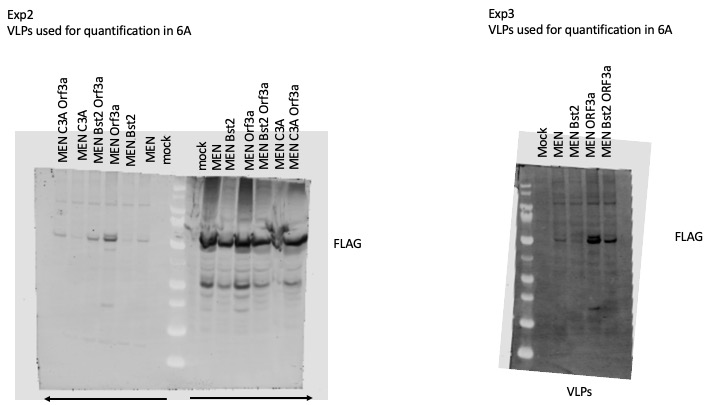

Supplement: Supplementary file 10 — Source Data for Figure 6 [file EMBR-24-e57224-s007.zip › SD Figure 6/6A/Figure 6A Part E.jpeg]

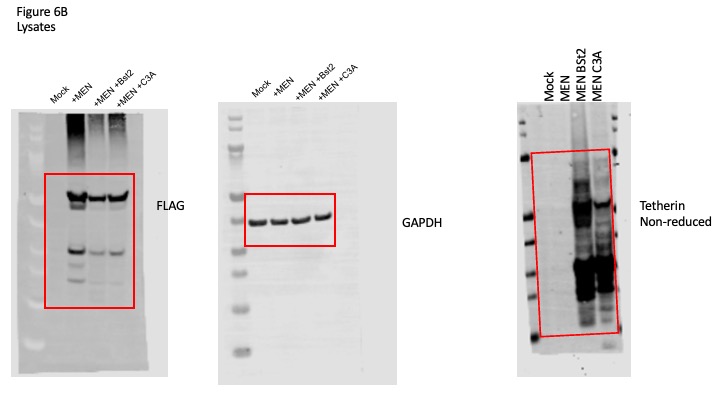

Supplement: Supplementary file 10 — Source Data for Figure 6 [file EMBR-24-e57224-s007.zip › SD Figure 6/6B/Figure 6B.jpeg]

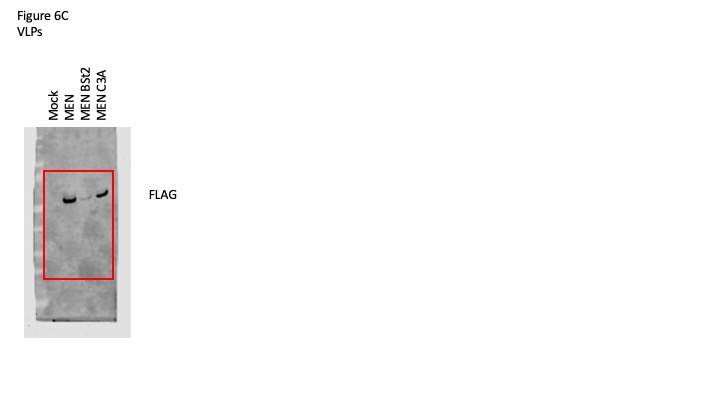

Supplement: Supplementary file 10 — Source Data for Figure 6 [file EMBR-24-e57224-s007.zip › SD Figure 6/6C/Figure 6C.jpeg]
